# Vine nitrogen status and volatile thiols and their precursors from plot to transcriptome level

**DOI:** 10.1186/s12870-016-0836-y

**Published:** 2016-08-08

**Authors:** Pierre Helwi, Sabine Guillaumie, Cécile Thibon, Céline Keime, Aude Habran, Ghislaine Hilbert, Eric Gomes, Philippe Darriet, Serge Delrot, Cornelis van Leeuwen

**Affiliations:** 1 Univ. de Bordeaux, Institut des Sciences de la Vigne et du Vin (ISVV), Ecophysiologie et Génomique Fonctionnelle de la Vigne (EGFV), UMR 1287, 33140 Villenave d’Ornon, France; 2 Bordeaux Sciences Agro, Institut des Sciences de la Vigne et du Vin (ISVV), Ecophysiologie et Génomique Fonctionnelle de la Vigne (EGFV), UMR 1287, 33140 Villenave d’Ornon, France; 3 INRA, Institut des Sciences de la Vigne et du Vin (ISVV), Ecophysiologie et Génomique Fonctionnelle de la Vigne (EGFV), UMR 1287, 33140 Villenave d’Ornon, France; 4 Univ. de Bordeaux, Institut des Sciences de la Vigne et du Vin (ISVV), Unité de recherche Œnologie, EA 4577, 33140 Villenave d’Ornon, France; 5 INRA, Institut des Sciences de la Vigne et du Vin (ISVV), USC 1366 Œnologie, 33140 Villenave d’Ornon, France; 6 Univ. de Strasbourg, Institut de Génétique et de Biologie Moléculaire et Cellulaire (IBGMC), Institut National de la Santé et de la Recherche Médicale U 964, Centre National de Recherche Scientifique UMR 7104, 67404 Illkirch, France

**Keywords:** *Vitis vinifera*, Nitrogen, Volatile thiols, 3-sulfanylhexan-1-ol, 4-methyl-4-sulfanylpentan-2-one, Glutathionylated precursors, Cysteinylated precursors, *GST*, *GGT*

## Abstract

**Background:**

Volatile thiols largely contribute to the organoleptic characteristics and typicity of Sauvignon blanc wines. Among this family of odorous compounds, 3-sulfanylhexan-1-ol (3SH) and 4-methyl-4-sulfanylpentan-2-one (4MSP) have a major impact on wine flavor. These thiols are formed during alcoholic fermentation by the yeast from odorless, non-volatile precursors found in the berries and the must. The present study investigates the effects of vine nitrogen (N) status on 3SH and 4MSP content in Sauvignon blanc wine and on the glutathionylated and cysteinylated precursors of 3SH (Glut-3SH and Cys-3SH) in the berries and the must. This is paralleled by a RNA-seq analysis of gene expression in the berries. The impact of N supply on the expression of the glutathione-*S*-transferase 3 and 4 (*VviGST3* and *VviGST4*) and the γ-glutamyltranspeptidase (*VviGGT*), considered as key genes in their biosynthesis, was also evaluated.

**Results:**

N supply (N100 treatment) increased the 3SH content in wine while no effect was noticed on 4MSP level. Furthermore, N supply increased Glut-3SH levels in grape berries at late berry ripening stages, and this effect was highly significant in must at harvest. No significant effect of N addition was noticed on Cys-3SH concentration. The transcript abundance of the glutathione-*S*-transferases *VviGST3* and *VviGST4* and the γ-glutamyltranspeptidase (*VviGGT*), were similar between the control and the N100 treatment. New candidate genes which might be implicated in the biosynthetic pathway of 3SH precursors were identified by whole transcriptome shotgun sequencing (RNA-seq).

**Conclusions:**

High vine N status has a positive effect on 3SH content in wine through an increase of Glut-3SH levels in grape berries and must. Candidate GSTs and glutathione-*S*-conjugates type transporters involved in this stimulation were identified by RNA-seq analysis.

**Electronic supplementary material:**

The online version of this article (doi:10.1186/s12870-016-0836-y) contains supplementary material, which is available to authorized users.

## Background

Aroma compounds contribute to a large extent to the organoleptic characteristics and typicity of wine. Slight differences in the concentrations of these volatile molecules create diverse wine aroma profiles [1, 2]. Wine aromas can be classified into three main groups according to their origin: “varietal” aromas specific for grape varieties, “winemaking” aromas produced during the fermentations and “mature” aromas developed during aging [3].

Varietal aroma compounds play a major role in the oenological potential of the wine. In addition to grape variety, their concentrations also depend on soil and climate [4]. Some varietal aroma compounds like pyrazines [5, 6], terpenols [7] and rotundone [8] exist as free compounds that are directly perceivable by the olfactory receptors. Others are present as non-volatile and odorless precursors in the grape berry and their cleavage into fragrant compounds occurs during winemaking and/or aging. Among the latter, major contributors to wine aromas are volatile thiols, monoterpènes, C13-norisoprenoids, dimethylsulfide, volatile phenols, C6 compounds and furaneol [9, 10].

**Fig. 1:**
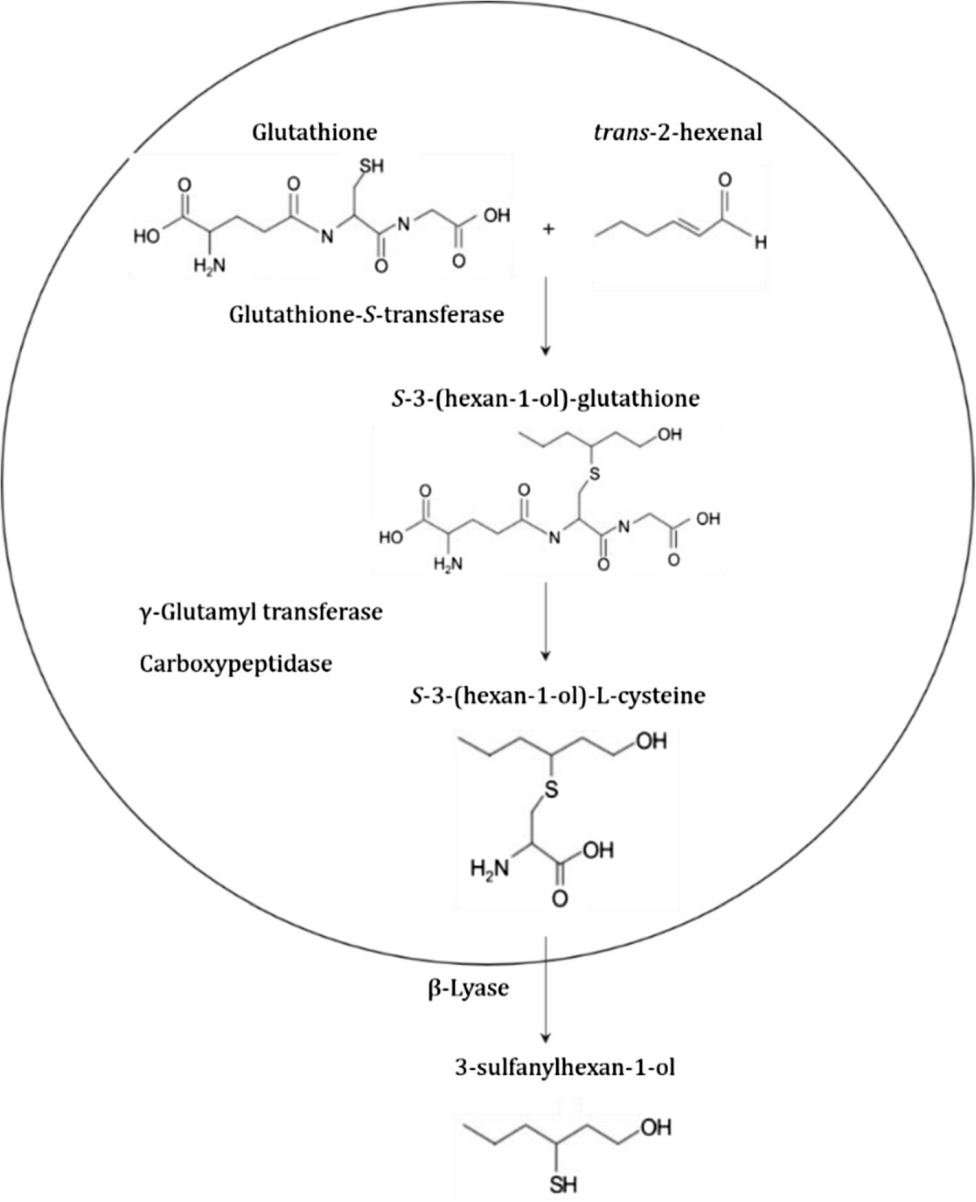
Hypothetical pathway of the glutathionylated precursor (Glut-3SH) and cysteinylated precursor (Cys-3SH) of 3SH in grape berries as described by Kobayashi et al. and Thibon et al. [21, 22]. In the berry, illustrated as a circle, Glut-3SH derives from Glut-3SH-al, which is formed by the combination of the glutathione (GSH) with the *trans*-2-hexenal. In the must, the production of 3-sulfanylhexan-1-ol (3SH) occurs during the alcoholic fermentation by the yeast

Volatile thiols represent a large family of molecules that contribute positively or negatively to the aromatic potential of the wines [11]. Many odorous thiols were identified in the early 1990s in Sauvignon blanc wines [12, 13]. Subsequently, these thiols were identified in a large panel of cultivars like Semillon, Riesling, Pinot gris and Colombard [14]. Among them, 4-methyl-4-sulfanylpentan-2-one (4MSP) and 3-sulfanylhexan-1-ol (3SH) identified by Darriet et al. (1995) and Tominaga et al. (1998) respectively, play an important role in the aroma of these varieties [12, 13]. In Sauvignon blanc wines, the 4MSP has a strong box-tree odor and the 3SH has a smell of grapefruit or passion fruit. The latter can be transformed during the alcoholic fermentation into its acetate reminiscent to boxwood, grapefruit zest and passion fruit [14]. The 4MSP and 3SH are not present in berries and musts, but they are generated during the alcoholic fermentation from non-volatile and odorless berry precursors. The known forms of these precursors in berries are *S*-conjugates to glutathione or to cysteine: *S*-4-(4-methylpentan-2-one)-L-glutathione (Glut-4MSP) and *S*-4-(4-methylpentan-2-one)-L-cysteine (Cys-4MSP) for the 4MSP respectively and *S*-3-(hexan-1-ol)-glutathione (Glut-3SH) and *S*-3-(hexan-1-ol)-cysteine (Cys-3SH) for the 3SH respectively [15–18]. The release of the volatile thiol from their precursors occurs during the fermentation by a β-lyase-like activity of *Saccharomyces cerevisiae* that induces the cleavage of carbon-sulfur bonds [15–17, 19, 20]. The biosynthetic pathways of these precursors in grape are still far from being fully understood, although some results have been published with regards to 3SH precursors. Peyrot des Gachons [16] assumed that the cysteinylated precursor of 3SH derives from the catabolism of the glutathionylated precursor. However this reaction requires the presence of two enzymes: a γ-glutamyltranspeptidase (VviGGT) which catalyzes the elimination of the glutamic acid, and a carboxypeptidase responsible for removing the glycine to produce the Cys-3SH. Recent studies also showed the formation of Cys-3SH in cell cultures of *Vitis vinifera* from Glut-3SH [21, 22]. The latter derives from an intermediate form, Glut-3SH-al, which is formed by the combination of the glutathione (GSH) with the *trans*-2-hexenal [23, 24]. This step could happen spontaneously and/or by the action of a glutathione-*S*-transferase enzyme (VviGST, Fig. 1) [23, 25, 26]. Kobayashi et al. described three genes in Sauvignon blanc berries putatively implicated in the synthesis of 3SH precursors: *VviGST3*, *VviGST4* and *VviGGT* [27].

In Sauvignon blanc berries and during ripening, the accumulation and the concentration of 4MSP and 3SH precursors largely depend on environmental factors like climate and soil composition [4, 28]. Among the nutrients vines absorbed from the soil, nitrogen (N) greatly influences the content of 4MSP and 3SH in wine and its precursors in grapes [4, 28, 29]. N supply enhances their synthesis and their accumulation in Sauvignon blanc grape berries, musts and wines [28, 29]. However, an increase of precursors levels in grapes and musts is not always correlated with an increase of 3SH content in wine due to nitrogen catabolite repression or other factors (phenols content, sugar level, pH…) during fermentation [30].

In the present study, the effect of vine N status on 4MSP and 3SH content in wines was confirmed. The impact of N status on the accumulation of Glut-3SH and Cys-3SH in grape berries during ripening and in musts was explored. Moreover *VviGST3*, *VviGST4* and *VviGGT* expression profiling was evaluated to determine their responses to N addition and their implication in 3SH synthesis. Furthermore, an identification of potential candidate genes that might be involved in the biosynthetic pathway of 3SH was performed by whole transcriptome shotgun sequencing (RNA-seq).

The objective of this study was to gain a better understanding on 3SH synthesis through monitoring of 3SH precursors genesis in grape berries and musts, 3SH liberation in wine and expression profiling of candidate genes during key stages in grape ripening under different levels of vine N status.

## Methods

### Location, vine material and experimental set up

Experiments were set up in commercial vineyards located in Pessac-Léognan area, Bordeaux, France (Château Couhins) and Sancerre area, France (Domaine Fontaine-Audon). Planting density was 6667 vines ha^−1^ in Château Couhins and 7000 vines ha^−1^ at domaine Fontaine-Audon. The study was performed on Sauvignon blanc vines (*Vitis vinifera* L.) in 2013 and 2014 (clone 316 grafted on Fercal in 2013 and clone 108 grafted on 161–49 C in 2014 in Bordeaux; clone 905 grafted on 3309 C in 2013 and 2014 in Sancerre). Experimental plots were selected for their low N status in previous years (Yeast Available Nitrogen (YAN) < 100 mg L^−1^). On each plot, two treatments were compared: control without fertilization and soil N100: 100 kg per hectare of nitrogen applied to the soil in two applications (bud break and flowering). Each treatment was repeated by means of four randomized replicates of ten vines each. Ammonium nitrate (NH_4_NO_3_) containing 33 % of N was used as fertilizer. Irrigation was managed during the whole season to avoid possible water deficit. In both vineyards, vines were double Guyot trained and all viticultural practices were identical for both modalities. No leaf removal or cluster and shoot thinning were carried out. Approximately 100 fresh berries were randomly collected per treatment from each block at three different developmental stages: mid-veraison (v), mid-ripening (28 days after mid-veraison; v + 28) and ripeness (35 days after mid-veraison; v + 35). Mid-veraison was determined as the time when 50 % of the berries were soft. Berries were cut at the pedicel without wounding berry skin and were frozen immediately in liquid nitrogen, ground and stored at − 80 °C.

### Vine water status

Vine water status was monitored during the season by measuring the leaf water potential with a pressure chamber [31]. Measurements were carried out at midday on three primary leaves per block covered with an opaque plastic bag 1 h prior to measurement [32]. Irrigation was implemented when leaf water potential was close to − 1 MPa, in order to avoid vine water deficit stress.

### Vine vigor

Vine vigor was assessed by measuring primary and secondary leaf areas and yield at harvest. To determine leaf areas, a correlation curve was established between the length of primary and secondary shoots and their corresponding leaf area using a LI-3100 LICOR leaf area meter (Lincoln, Nebraska, USA). Subsequently, the length of all primary and secondary shoots of two vines per replicate was measured and primary and secondary leaf areas were deduced from the correlation obtained between shoot length and shoot leaf area, according to Mabrouk and Carbonneau [33]. Yield was determined for each elementary plot at harvest by weighing all grape bunches of ten vines per replicate.

### Vine and berry nitrogen status

N-tester measurements (N-tester, Norsk Hydro, 00-12 Oslo, Norway) and Yeast Available Nitrogen (YAN) were used to assess vine N status [34]. N-tester measures leaf blade color intensity which is in relation with the chlorophyll concentration and the N status. Measurements were performed on thirty primary leaves in the bunch zone across each block in order to obtain an average value representing vine N status [34, 35]. YAN was assessed in grape juice obtained by pressing approximately 100 berries collected at harvest stage. The juice was analyzed with a Fourier Transform Infra-Red spectrometer (FTIR, WineScan FOSS®, FRANCE, 92000 Nanterre) [36].

### Berry and must composition

Harvest in Bordeaux vineyard occurred on September 23 in 2013 and on September 08 in 2014. In Sancerre, these dates were respectively October 10 in 2013 and September 25 in 2014. One day prior to these dates, approximately 25 berries were collected in liquid nitrogen for primary metabolite analyses and 100 berries were sampled and pressed to obtain grape must. Primary metabolites of berries were extracted from 250 mg of frozen berry material ground to powder, with 80 % ethanol at 80 °C for 15 min followed by two extractions with respectively 50 % ethanol and ultrapure water. Glucose and fructose were assessed using an enzymatic method (glucose/fructose kit from BioSenTec, BioSenTec, F-31000 Toulouse, France). Malic acid was measured using a colorimetric method (Bran and Luebbe TRAACS 800 autoanalyzer, 22844 Norderstedt, Germany).

Grape must was obtained by pressing 100 berries. After centrifugation, it was analyzed using a WineScan™ Auto analyzer (WineScan FOSS®, FRANCE, 92000 Nanterre) according to Destrac et al. [36]. Sugar, total acidity, pH and malic acid contents were measured twice on each sample through two successive determinations.

### Microvinification

Microvinifications were conducted on Sauvignon blanc from each plot in Bordeaux and Sancerre as described [6]. In Bordeaux, one kilogram of grapes were pressed manually and in Sancerre, 20 kg of grapes were pressed with a small wine press (Socma, 11100 Narbonne, France). In Bordeaux and Sancerre, must was inoculated with X5 yeast strain (ZYMAFLORE X5 - Laffort, final concentration of 20 mg mL^−1^) and fermentations were carried on 100 mL and 5 L of must respectively at 20 °C.

### Quantification of 4MSP and 3SH in wines

For 50 mL of centrifuged wine, 2 mM of *p*-hydroxymercuribenzoate diluted in 0.1 M Tris and 50 μL of internal standard mix (6-sulfanylhexanol: 6SH, 30 μmol L^−1^, and 4-methoxy-2-methyl-2-sulfanylbutan: MMSB, 30 μmol L^−1^) were added successively. Sample pH was adjusted to 7.0 (with concentrated NaOH solution), and then purified on an ion exchange resin (Dowex 1, Sigma; 1x2-100) previously activated for 15 min with HCl (0.1 M) and washed with water until its pH reached a value of 5.0. The resin was washed with an acetate buffer (50 mL, 0.1 M, pH 7.0) and subsequently thiols were eluted by a cysteine buffer (50 mL, 10 g L^−1^, pH 7). The flow-through was collected, mixed with 500 μL of ethyl acetate and volatile thiols were extracted twice with dichloromethane (4 mL than 3 mL). The organic phase containing thiols was dried with anhydrous sodium sulfate and then concentrated to obtain a final volume of 50 μL. Samples were analyzed by gas chromatography coupled to mass spectrometry (GC-MS) as described [37].

### Extraction and quantification of 3SH precursors from berries and grape musts

The extraction and the quantification of 3SH precursors in grape berries and musts was performed as described [38]. In brief, the purification was carried on a mix of 1 mL of grape juice obtained by defrosting of 2 g of frozen berry powder in the presence of sulfur dioxide (200 mg L^−1^) or directly from grape must, 1 mL of water and a final concentration of 50 μg L^−1^ of the internal standard solution containing a deuterated form of the glutathionylated *S*-conjugate of 3SH ((3-*S*-hexan-1-ol)-glutathione-d_3_) percolated through a SPE columns (LC-18 500 mg 6 mL, Supelco France, Saint Germain-Laye, France). Precursors were eluted with 3 mL of methanol/water (30/70; v/v) in hemolysis tubes. The flow-through was subsequently evaporated and residues were dissolved in 700 μL of aqueous formic acid solution (0.1 %). Quantification was done using an Accela UHPLC (Thermo Fisher Scientific) connected in series to an Exactive (Thermo Fisher Scientific, Bremen, Germany) mass spectrometer equipped with a heated ESI ion source (LC-ESI-MS). The separation was performed on a Synchronis aQ column (100 × 2.1 mm i.d., 1.7 μm, Synchronis aQ, Thermo Scientific, Bremen, Germany) with a flow rate of 300 μL min^−1^ of solvent A (0.1 % aqueous formic acid) and solvent B (0.1 % formic acid in acetonitrile). The ion source was operated in the positive ion mode at 3.5 kV.

### RNA extraction and gene expression analysis

Total RNAs were isolated according to Reid et al. [39] from 1 g of grounded frozen berries from Bordeaux in 2013 and 2014. Traces of genomic DNA were removed by a DNAse I treatment according to the manufacturer’s instructions (Ambion TURBO DNA-free DNase, Life Technologies). RNAs were quantified using a Nanodrop 2000c spectrophotometer (Thermo Scientific) and checked for integrity on an 1.8 % agarose gel. For cDNA synthesis, 1.5 μg of total RNA were reverse transcribed with OligodT primers in an 20 μL reaction mixture using SuperScript III reverse transcriptase (Invitrogen) according to the manufacturer’s instructions.

Relative transcript quantification of *VviGSTs, VviGGT* and *VviNiR *genes was achieved by quantitative real-time PCR using a CFX96 Real-Time PCR Detection system (Bio-Rad). PCR conditions and specific oligonucleotide primer pairs were those from Kobayashi et al. [27] and Guillaumie et al. [40]. The amplification efficiencies were determined by serial dilutions and normalized expression of each gene was calculated using the Bio-Rad CFX Manager software. Amplifications of the *VviGAPDH* and *VviActin* genes were used for normalization by comparing the cycle threshold of the target gene with those of standard genes. All experiments were performed with three biological replicates and two technical replicates.

### RNA-seq library construction and sequencing

Grape berry mRNA was purified using oligo (dT) magnetic beads and fragmented using divalent cations at 94 °C for 8 min. mRNA was cleaved into short fragments of about 200 bp and double strand cDNA was synthetized using random primers and DNA polymerase I. The double stranded cDNA fragments were blunted using T4 DNA polymerase, Klenow DNA polymerase and T4 PNK. A single ‘A’ nucleotide was added to the 3’ ends of the blunt DNA fragments using a Klenow fragment enzyme. Sequencing adaptors were ligated to the fragments that were enriched by PCR amplification (TruSeq™ RNA Sample Preparation v2 kit, Illumina Inc.). Surplus PCR primers were removed by purification using AMPure XP beads (Agencourt Biosciences Corporation). DNA libraries were checked for quality and quantified using 2100 Bioanalyzer (Agilent). The grape berry cDNA library products were ready for sequencing analysis via Illumina Hiseq 2500. The RNA-seq library construction and sequencing was realized by the platform “Biopuces et Sequençage” in Strasbourg, France.

### Analysis of Illumina reads

Reads were mapped onto the 12X assembly of the *Vitis vinifera* genome (http://genomes.cribi.unipd.it/DATA/GENOME_12X/Genome12X.tar.gz), using TopHat v2.0.10 [41] and the Bowtie2 v2.1.0 aligner [42]. Only uniquely aligned reads have been retained for analysis. Quantification of gene expression was performed using HTSeq v0.5.4p3 [43] with the intersection nonempty mode and CRIBI V1 annotations. The gene expression level was calculated by using RPKM (reads per exon kilo base per million mapped sequence reads).

Statistical analysis was performed using the method proposed by Love et al. [44] and implemented in the DESeq2 Bioconductor library (DESeq2 v1.0.19). Adjustment for multiple testing was performed with the Benjamini and Hochberg method [45]. The log_2_(Fold-Change) (LFC) was estimated using the method proposed by Love et al. [44]. Genes of interest were defined by their bin code from Ath_AGI_TAIR9_Jan2010 using MapMan interface [46]. Best identity descriptions of genes were defined from annotation results available on the CRIBI website (http://genomes.cribi.unipd.it/DATA) and from Ath_AGI_TAIR9_Jan2010 using MapMan interface. Differentially expressed genes with a *p* value ≤ 0.05 and an absolute value of LFC ratio ≥ 0.6 were used as the thresholds to judge the significance of gene expression difference. Three biological replicates for each condition were included in this analysis. The RNA-seq dataset was deposited in the Gene Expression Omnibus (GEO), National Center for Biotechnology Information (NCBI) under the accession number GSE77895.

### Statistical analysis

Statistical analysis were conducted using the statistical package of the R software (R Development Core team, 2010). All the data are expressed as the mean average ± standard error (SE) from four biological replicates for metabolites measurements and from three biological replicates and two technical replicates for gene expression profiling. Statistical significance was analyzed with Student’s *t* test. A value of *p* value ≤ 0.05 was considered to indicate statistical significance.

## Results

### Vine water status

**Fig. 2:**
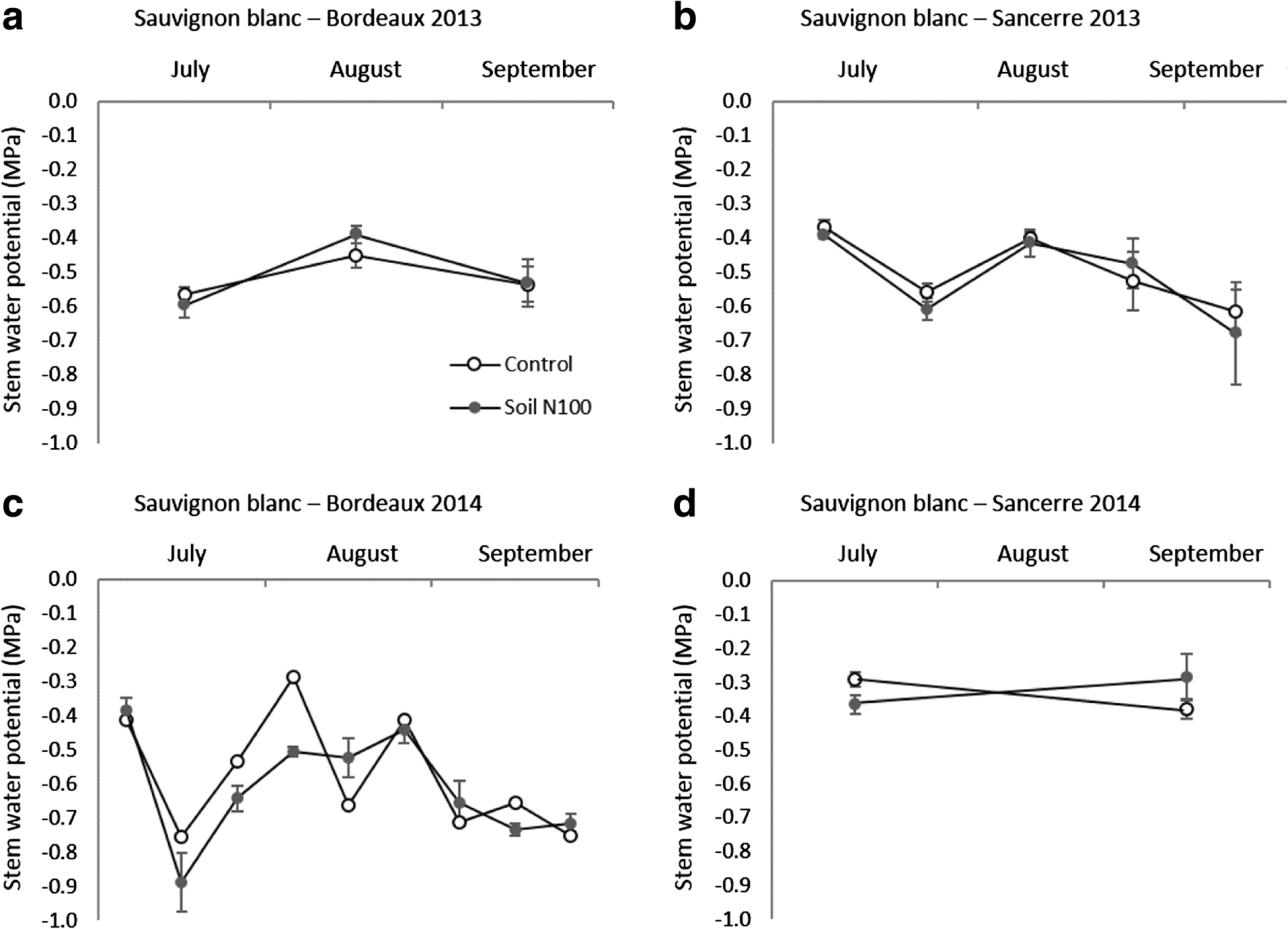
Stem water potential (MPa) as an indicator of vine water status, measured in Bordeaux (**a** and **c**) and Sancerre (**b** and **d**) in 2013 and 2014. All data are presented as the mean of four biological replicates. Error bars indicate Standard Error (SE)

The aim of this experimentation was to study the direct effect of N status on aroma potential without interference of water deficit. Irrigation was implemented to avoid water deficit stress. Vine water status was assessed during both seasons from July through September, by measuring stem water potential using a pressure chamber [32]. Stem water potential values for both vineyards are shown in Fig. 2.

In 2013, stem water potential values ranged between − 0.7 MPa and − 0.3 MPa and so did not exceed the threshold for water deficit of − 1 MPa. In 2014, stem water potential values ranged between − 0.95 MPa and − 0.2 MPa in Bordeaux and − 0.45 MPa and − 0.3 MPa in Sancerre. Hence, vines did not face water deficit, which allowed us to study the influence of N fertilization without interference with water stress.

### Vine vigor

N supply could enhance vegetative growth and increase leaf area and yield. To limit a possible impact of N supply on vine vigor, the fertilization was fractionated into two doses for the soil N100 treatment and it was applied late in the season (at budbreak and at bloom). Vine vigor was estimated by measuring primary and secondary leaf areas at shoot growth cessation and by yield assessment at harvest (Table 1).

**Table 1:** The influence of N supply on primary and secondary leaf areas (m^2^ vine^−1^) measured at shoot growth cessation and yield (kg vine^−1^) determined at harvest in 2013 and 2014

	Bordeaux			Sancerre		
	Primary leaf area	Secondary leaf area	Yield	Primary leaf area	Secondary leaf area	Yield
2013						
Control	1.11 ^a^	1.02 ^a^	1.18 ^a^	1.59 ^a^	0.59 ^a^	1.44 ^a^
Soil N100	1.18 ^a^	1.30 ^a^	1.24 ^a^	1.69 ^a^	0.95 ^b^	1.47 ^a^
2014						
Control	1.06 ^a^	1.21 ^a^	1.24 ^a^	1.63 ^a^	0.56 ^a^	2.57 ^a^
Soil N100	1.18 ^a^	1.43 ^a^	1.10 ^a^	1.76 ^b^	1.37 ^b^	2.39 ^a^

Values are means of four replicates. In case of significant differences between the two treatments, different letters within the same parameter indicate significant differences. Statistical significance was determined by Student’s *t* test (*p* value ≤ 0.05)

In Bordeaux, in both years, primary leaf areas for the control and for the soil N100 treatment were similar. No differences were recorded for secondary leaf area in this region either. In Sancerre, N supply did not influence primary leaf area in 2013 while it was slightly increased in 2014. Furthermore, a difference was observed for secondary leaf area in this region in both years. Soil N100 treatment developed greater leaf area compared to the control.

Yields at harvest were determined for both treatments in both vineyards. For each year and location, no significant differences of yield were observed between the N100 treatment and the control (Table 1).

Hence, vine N status did not modify vine vigor in the Bordeaux experiment in both years. In the Sancerre experiment, vine vigor was slightly increased, as shown by a greater secondary leaf area in the soil N100 treatment compared to the control in both years.

### Vine and berry nitrogen status

**Fig. 3:**
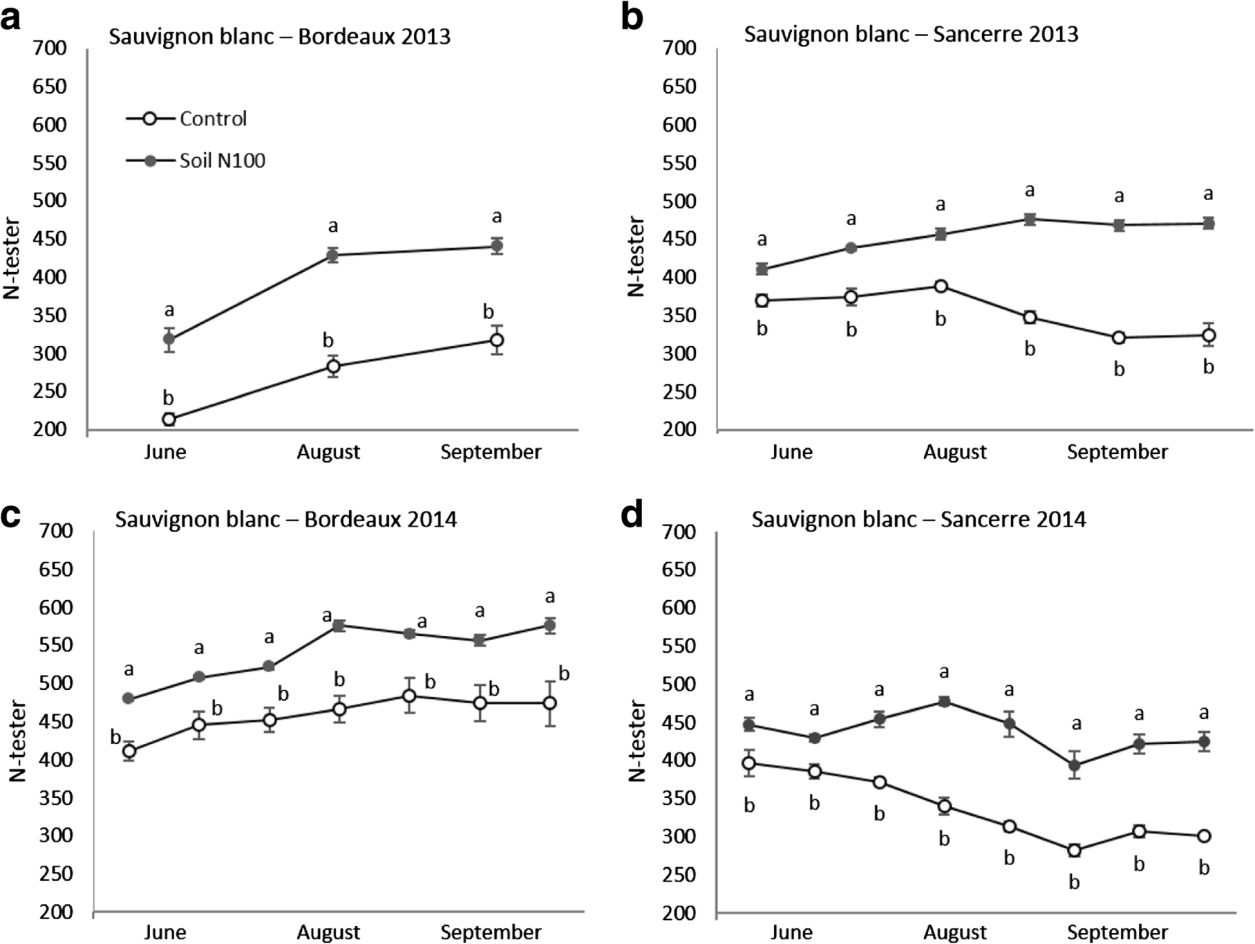
N-tester measurements, as an indicator of vine nitrogen status in Bordeaux (**a** and **c**) and Sancerre (**b** and **d**) vineyards in 2013 and 2014. This device measures the intensity of the leaf blade color which is in relation with chlorophyll content. All data are presented as the mean of four biological replicates. Different letters indicate significant differences. Error bars indicate Standard Error (SE). Statistical significance was determined by Student’s *t* test (*p* value ≤ 0.05)

Vine and berry N status were assessed in order to check if this nutrient was correctly assimilated in the fertilized vines. Vine N status determined by N-tester measurements was higher for soil N100 treatment compared to the control during the whole season in both regions and years (Fig. 3). Differences were statistically significant for all measurements.

**Fig. 4:**
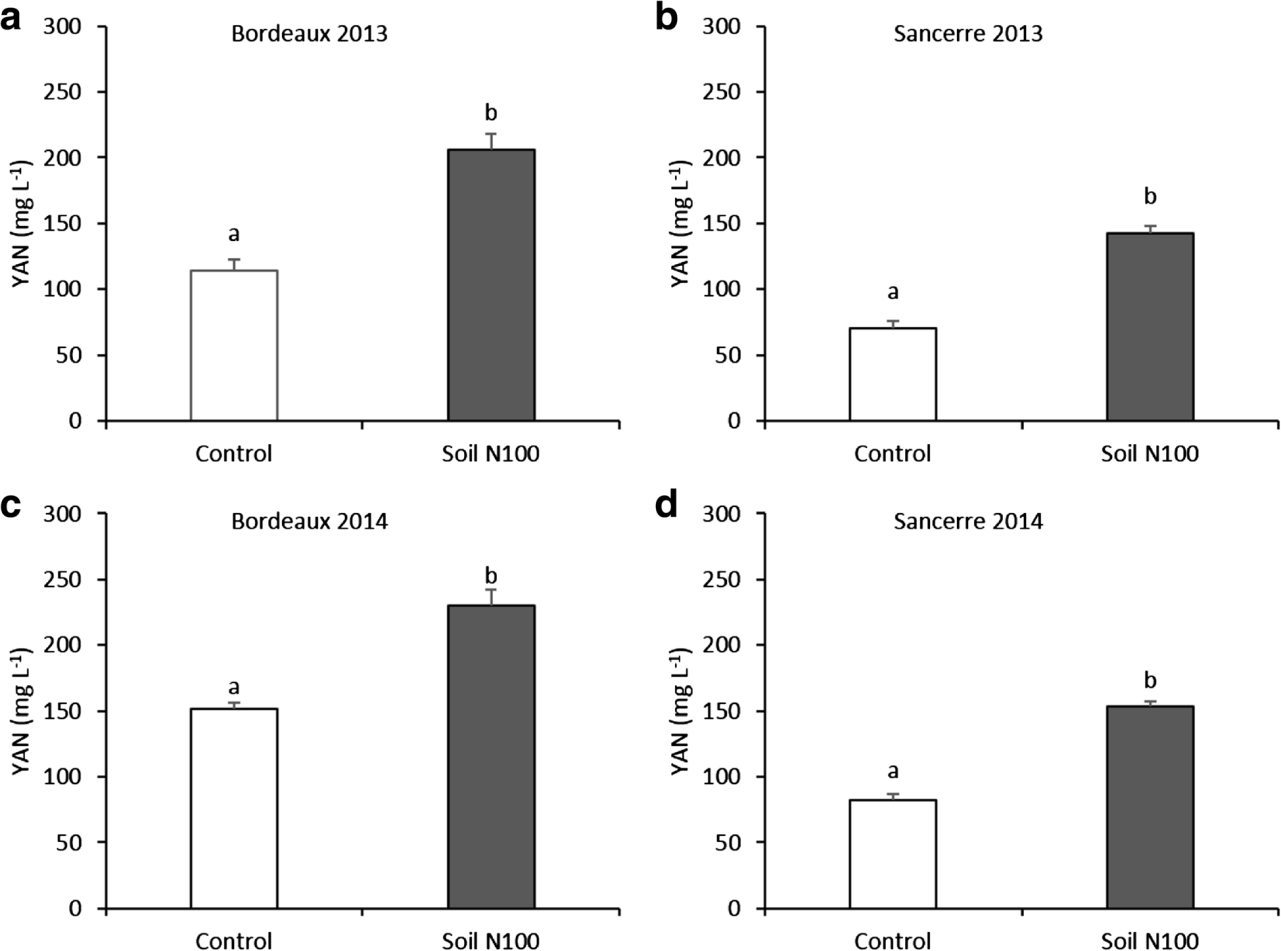
Yeast Available Nitrogen (YAN) determined for each plot in 2013 and 2014 prior to commercial harvest (mg L^−1^). All data are presented as mean of four biological replicates. Different letters indicate significant differences. Error bars indicate Standard Error (SE). Statistical significance was determined by Student’s *t* test (*p* value ≤ 0.05). The white and grey bars represent the control and the soil N100 treatment respectively

Differences among treatments were confirmed by YAN measurements prior to commercial harvest. In both years and locations, YAN was always significantly higher in the N100 treatment compared to the control (Fig. 4).

### Grape berries maturity and must composition

The effect of N supply on Sauvignon blanc grape berry and must composition was investigated to determine if the rate of maturity of berries was affected by the fertilization (Table 2). Grape maturity was estimated by measuring berry weight, sugars (glucose and fructose) and malic acid content at ripeness.

**Table 2:** Nitrogen effect on berry weight (in gberry^−1^), sugars (glucose and fructose) and malic acid contents (in mg g^−1^ of fresh material) measured just prior to harvest

	Bordeaux				Sancerre			
	Berry weight	Glucose	Fructose	Malic acid	Berry weight	Glucose	Fructose	Malic acid
2013								
Control	1.62 ^a^	112.8 ^a^	111.1 ^a^	4.3 ^a^	1.49 ^a^	100.6 ^a^	96.4 ^a^	3.1 ^a^
Soil N100	1.79 ^b^	98.4 ^b^	97.2 ^b^	5.1 ^b^	1.59 ^a^	106.4 ^a^	105.4 ^a^	3.9 ^b^
2014								
Control	2.26 ^a^	82.9 ^a^	81.1 ^a^	3.4 ^a^	1.89 ^a^	82.2 ^a^	76.6 ^a^	4.2 ^a^
Soil N100	2.18 ^a^	79.1 ^a^	77.5 ^a^	4.4 ^b^	1.96 ^a^	83.9 ^a^	78.9 ^a^	4.6 ^b^

Values are means of four replicates. In case of significant differences between the two treatments, different letters within the same parameter indicate significant differences. Statistical significance was determined by Student’s *t* test (*p* value ≤ 0.05)

In the present study, vine N status did not effect berry weight except for Bordeaux in 2013 where berry weight was higher in the soil N100 treatment (Table 2). It should be noted that this difference, although statistically significant, is not relevant from an enological point of view. The concentration of berry sugars (glucose and fructose) were similar among the two treatments, except in Bordeaux in 2013 where a difference of approximately 15 mg L^−1^ of glucose and fructose was observed between the control and the fertilized modality. Malic acid was always lower in the control compared to N100 treatment. Differences ranged from 0.4 mg g^−1^ (Sancerre 2014) to 1.0 mg g^−1^ (Bordeaux 2014).

The effect of N addition on sugar and malic acid content, pH and acidity was also measured in grape must just prior to commercial harvest (Table 3). No effect of N fertilization was noticed on sugar level in Bordeaux in 2013 and Sancerre in 2014. A decrease of 16 g L^−1^ and 5 g L^−1^ in sugar content was observed in soil N100 treatment in Bordeaux in 2014 and Sancerre in 2013 respectively. pH was not impacted by the fertilization and only in Bordeaux in 2014 an increase of 0.6 g tartrate eq. L^−1^ in total acidity was observed. In Bordeaux in both years and in Sancerre in 2014, malic acid concentration was higher in berries harvested in soil N100 treatment. In Bordeaux, differences in malic acid level between the two treatments were in the order of 1.3 g L^−1^ and 0.7 g L^−1^ in 2013 and 2014 respectively while in Sancerre in 2014, an increase of 0.4 g L^−1^ was measured in the N100 treatment. Grape ripening was delayed by N fertilization, as shown by decreased sugar concentrations and/or increased malic acid concentrations. However, although these differences are statistically significant, they correspond to a delay in ripeness of only 2–4 days, depending on location and year. Difference did not seem great enough to justify different sampling dates for maturity for the control versus the soil N100 treatment.

**Table 3:** The effect of nitrogen addition on sugar level (g L^−1^), Total Acidity (g tartrate eq. L^−1^), pH and malic acid content (g L^−1^) in grape musts prior to harvest in 2013 and 2014

	Bordeaux				Sancerre			
	Sugar	Total Acidity	pH	Malic acid	Sugar	Total Acidity	pH	Malic acid
2013								
Control	193 ^a^	10.9 ^a^	2.99 ^a^	4.7 ^a^	205 ^a^	7.9 ^a^	3.17 ^a^	3.8 ^a^
Soil N100	174 ^a^	11.7 ^a^	3.01 ^a^	6.0 ^a^	190 ^b^	7.6 ^a^	3.20 ^a^	3.8 ^a^
2014								
Control	204 ^a^	10.8 ^a^	3.08 ^a^	5.1 ^a^	205 ^a^	9.0 ^a^	3.05 ^a^	4.6 ^a^
Soil N100	198 ^b^	11.4 ^b^	3.10 ^a^	5.8 ^b^	204 ^a^	9.4 ^a^	3.07 ^a^	5.1 ^b^

Values are means of four replicates. In case of significant differences between the two treatments, different letters within the same parameter indicate significant differences. Statistical significance was determined by Student’s *t* test (*p* value ≤ 0.05)

### Nitrogen effect on thiol level in wines

**Fig. 5:**
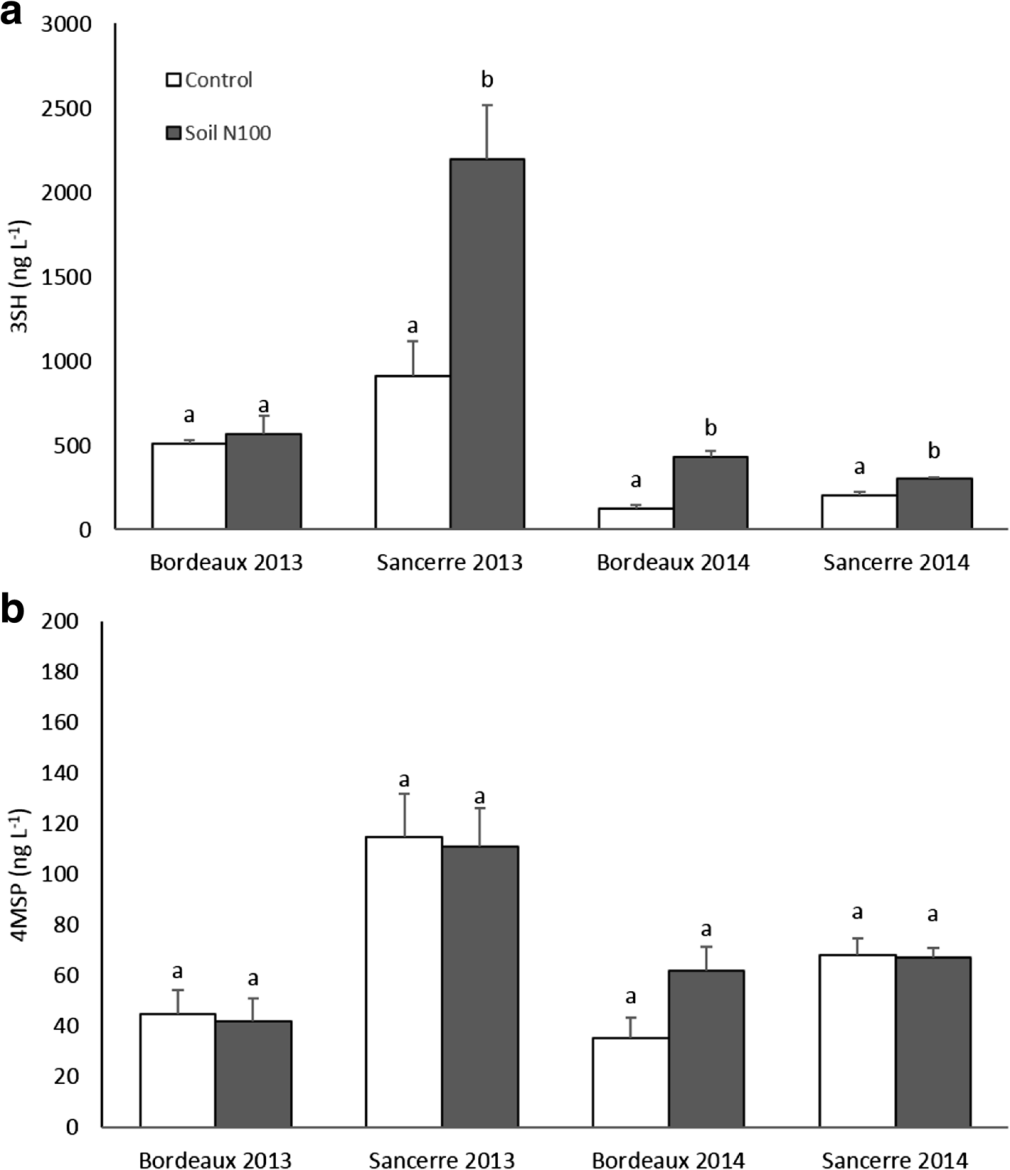
Effects of nitrogen supply on the level of (**a**) 3SH (ng L^−1^) and (**b**) 4MSP (ng L^−1^) in wines made by small-scale vinifications. All data are presented as mean of four biological replicates. Different letters indicate significant differences. Error bars indicate Standard Error (SE). Statistical significance was determined by Student’s *t* test (*p* value ≤ 0.05)

The effect of N fertilization on 3SH and 4MSP in Sauvignon blanc wine from Bordeaux and Sancerre was investigated in 2013 and 2014 (Fig. 5). Higher levels of 3SH were recorded in 2013 compared to 2014 in both vineyards. The concentration of 3SH was particularly high in Sancerre in 2013, where it reached 2198 ng L^−1^ for the soil N100 treatment versus 570 ng L^−1^ in Bordeaux for the same modality. As for 3SH, 4MSP level was the highest in Sancerre in 2013 where concentrations ranged between 110 and 115 ng L^−1^.

Concerning the impact of N supply on these volatile thiols, wines made from berries grown on fertilized vines had higher 3SH concentration than those made from berries grown on non-fertilized vines, although this difference was not significant in Bordeaux in 2013 (Fig. 5a). For example, compared to control plants, 3SH level in the soil N100 treatment was 2.5-fold higher in Sancerre in 2013 (2198 ng L^−1^ versus 911 ng L^−1^), 1.5-fold higher in Sancerre in 2014 (299 ng L^−1^ versus 201 ng L^−1^) and 3.5-fold higher in Bordeaux in 2014 (429 ng L^−1^ versus 123 ng L^−1^). No significant differences were observed on 4MSP levels between the two treatments in all combinations. Hence, vine N status does not influence 4MSP contents in Sauvignon blanc wines (Fig. 5b). For this reason, 4MSP and its precursors were not further investigated in this study.

### Nitrogen effect on Glut-3SH and Cys-3SH contents in grape berries

**Fig. 6:**
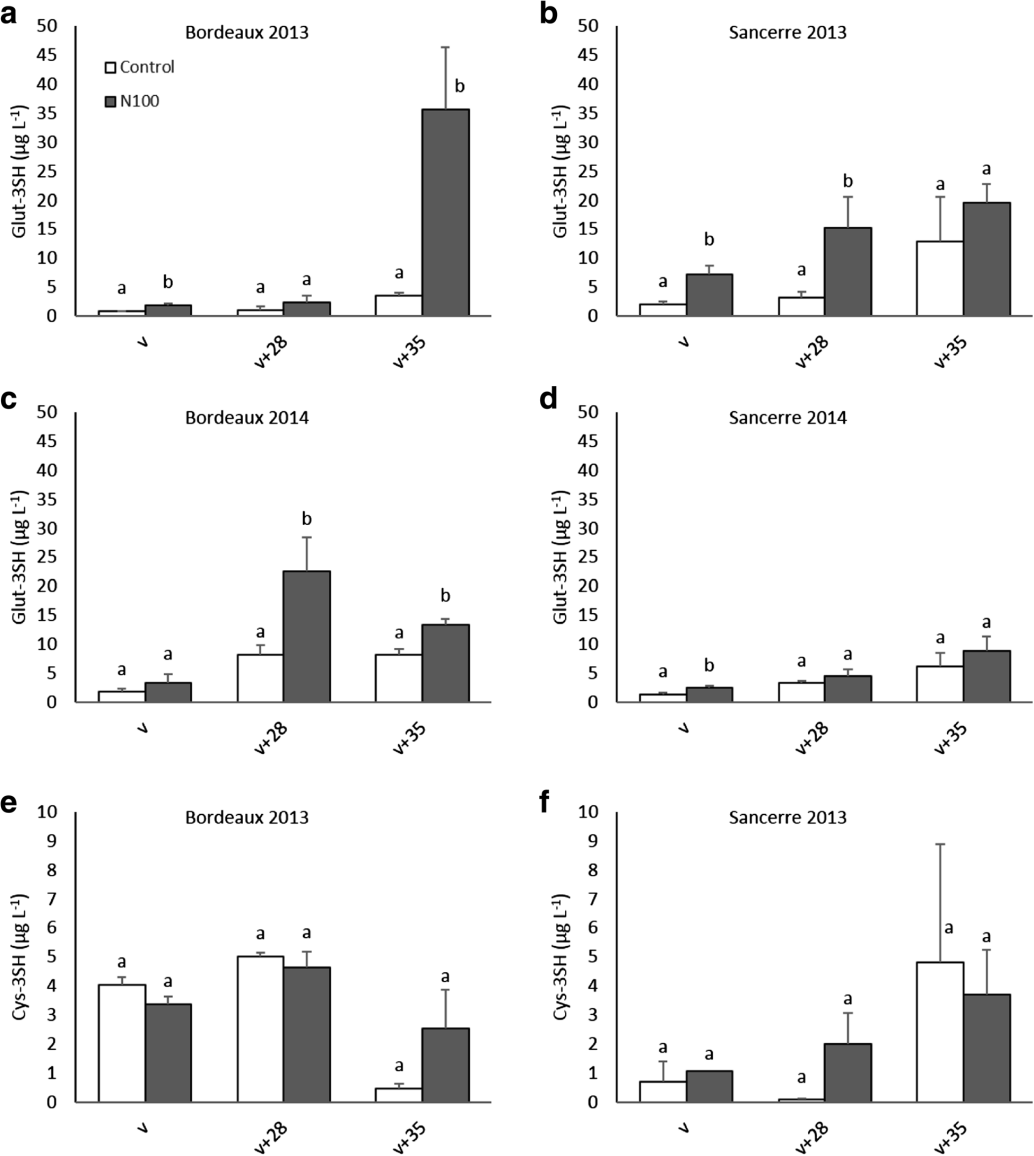
Effects of nitrogen supply on the amount of Glut-3SH (**a** - **d**) and Cys-3SH (**e** and **f**) (μg L^−1^) in grape berries at (v), (v + 28) and (v + 35). All data are presented as mean of four biological replicates. Different letters indicate significant differences. Error bars indicate Standard Error (SE). Statistical significance was determined by Student’s *t* test (*p* value ≤ 0.05)

To determine the effect of N supply on the glutathionylated (Glut-3SH) and the cysteinylated (Cys-3SH) precursors of 3SH in Sauvignon blanc berries, their content in the control and in the soil N100 treatment was monitored during three developmental stages, at mid-veraison (v), mid-ripening (v + 28) and ripeness (v + 35) (Fig. 6).

Glut-3SH concentration increased during berry ripening and reached a peak at (v + 35) except for Bordeaux in 2014 where the highest value was attained at (v + 28). Glut-3SH content was related to vine N status. Although statistically not always significant, Glut-3SH level was in general higher in the soil N100 treatment compared to the control in all combinations (Fig. 6a–d). For example in Bordeaux in 2013 at (v + 35), Glut-3SH content was 10-fold higher in the N100 treatment (35.75 μg L^−1^ versus 3.5 μg L^−1^ respectively, Fig. 6a) than in the control. In the same year in Sancerre at (v + 28), the content of the glutathionylated precursor in the N100 treatment was 5-fold higher than in the control (3.1 μg L^−1^ versus 15.2 μg L^−1^, Fig. 6b).

Cys-3SH was only detectable in Bordeaux and Sancerre in 2013 (Fig. 6e–f). In 2014 in both areas, its level was below the detection limits of the method (LOQ = 0.5 μg L^−1^). The concentration of Cys-3SH was relatively low and stable in the subsequent developmental stages. Moreover the content of this molecule was similar between the control and the soil N100 treatment, indicating that vine N status did not influence Cys-3SH content in Sauvignon blanc grape berries.

### Nitrogen effect on Glut-3SH and Cys-3SH contents in musts

**Fig. 7:**
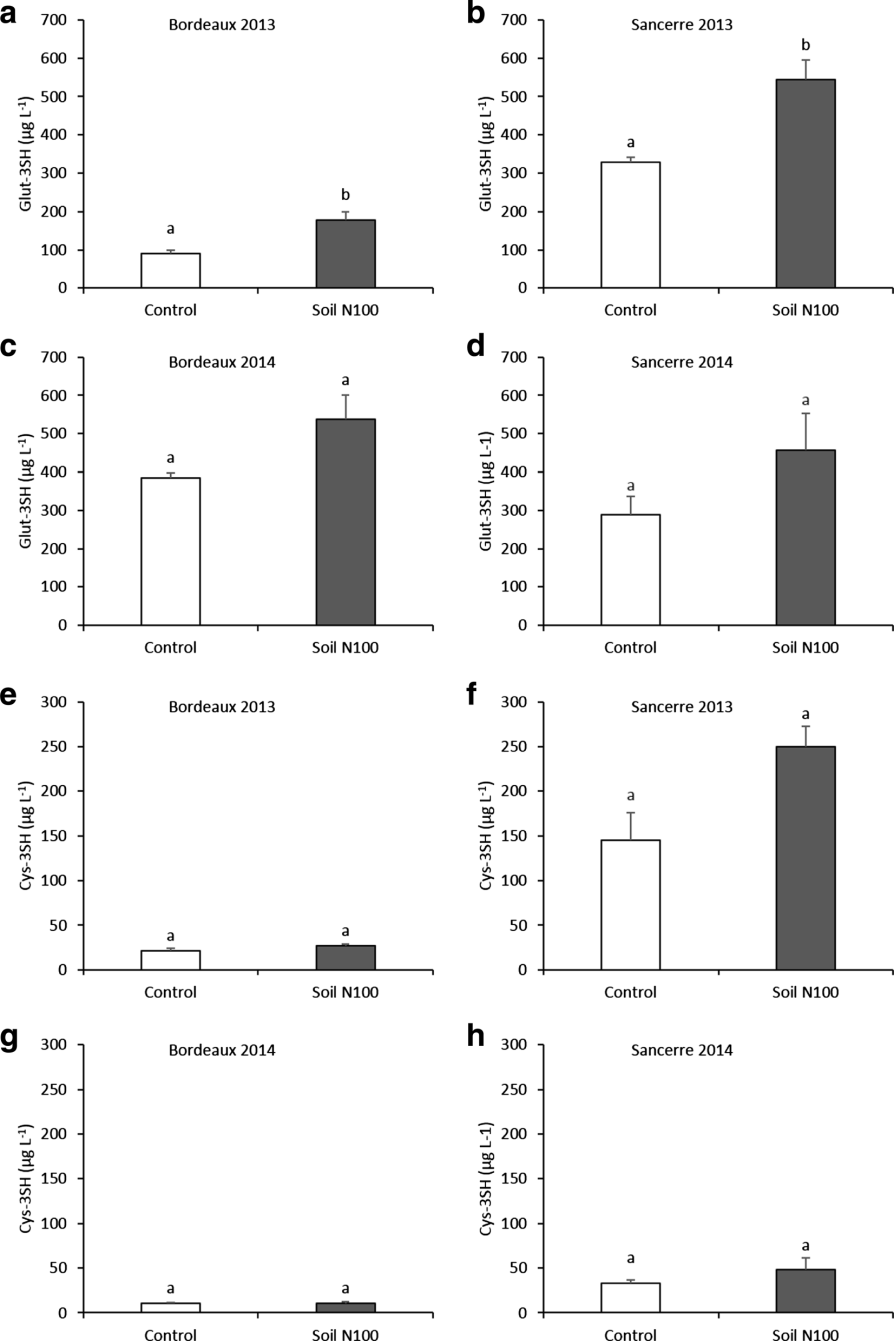
Effect of nitrogen supply on the level of Glut-3SH (**a** - **d**) and Cys-3SH (**e** - **h**) (μg L^−1^) in grape musts. All data are presented as mean of four biological replicates. Letters indicate significant differences. Error bars indicate Standard Error (SE). Statistical significance was determined by Student’s *t* test (*p* value ≤ 0.05)

Analyzing 3SH precursors in grape musts is important due to the fact that those precursors will be transformed in 3SH during the alcoholic fermentation. Several enological techniques like destemming, crushing, pressing and clarification may influence their level during must preparation before fermentation. The glutathionylated and the cysteinylated precursors of 3SH were assessed in Bordeaux and Sancerre Sauvignon blanc musts at harvest in 2013 and 2014 (Fig. 7). Compared to berries, concentrations of Glut-3SH and Cys-3SH were approximately 20-fold higher in the corresponding must demonstrating that important amounts of precursors in the must are synthesized during the pre-fermentation operations. Differences may also result from the technology of grape processing: immediate freezing in liquid nitrogen for grape berries versus pressing at room temperature to obtain grape must or from the way of grape pressing. Musts from Sancerre were in most cases richer in these precursors compared to musts from Bordeaux.

The fertilization led to a significant increase in the concentration of Glut-3SH in 2013 in both vineyards (Fig. 7a–b). Levels of Glut-3SH in the control were in order of 91 μg L^−1^ in Bordeaux and 328 μg L^−1^ in Sancerre and they increased to 178 μg L^−1^ and 544 μg L^−1^ respectively in the soil N100 treatment. In 2014 in both regions, only a trend for a higher Glut-3SH content was observed in the must from high N vines (Fig. 7c–d).

Levels of Cys-3SH were lower compared to Glut-3SH, which confirmed an earlier observation [47–49]. In both years, vine N status did not affect Cys-3SH level (Fig. 7e–h). In Bordeaux, Cys-3SH content ranged between 21 μg L^−1^ and 27 μg L^−1^ in 2013 while in 2014 it was in the order of 11 μg L^−1^. In Sancerre, Cys-3SH level was particularly high in 2013 where values ranged between 145 μg L^−1^ and 250 μg L^−1^. In the same year, the 3SH content in Sancerre wines was very high, which confirms a positive relation between the abundance of the precursors in the must and the abundance of the corresponding thiol in the wine. In 2014, the concentration of this molecule was in the same range as in Bordeaux with values comprised between 30 and 45 μg L^−1^.

These results showed that higher vine and berry N status increased the content of the glutathionylated precursor of 3SH in Sauvignon blanc grapes and musts while the concentration of the cysteinylated precursor was not affected.

### Gene expression from the 3SH pathway and their response to nitrogen fertilization

**Fig. 8:**
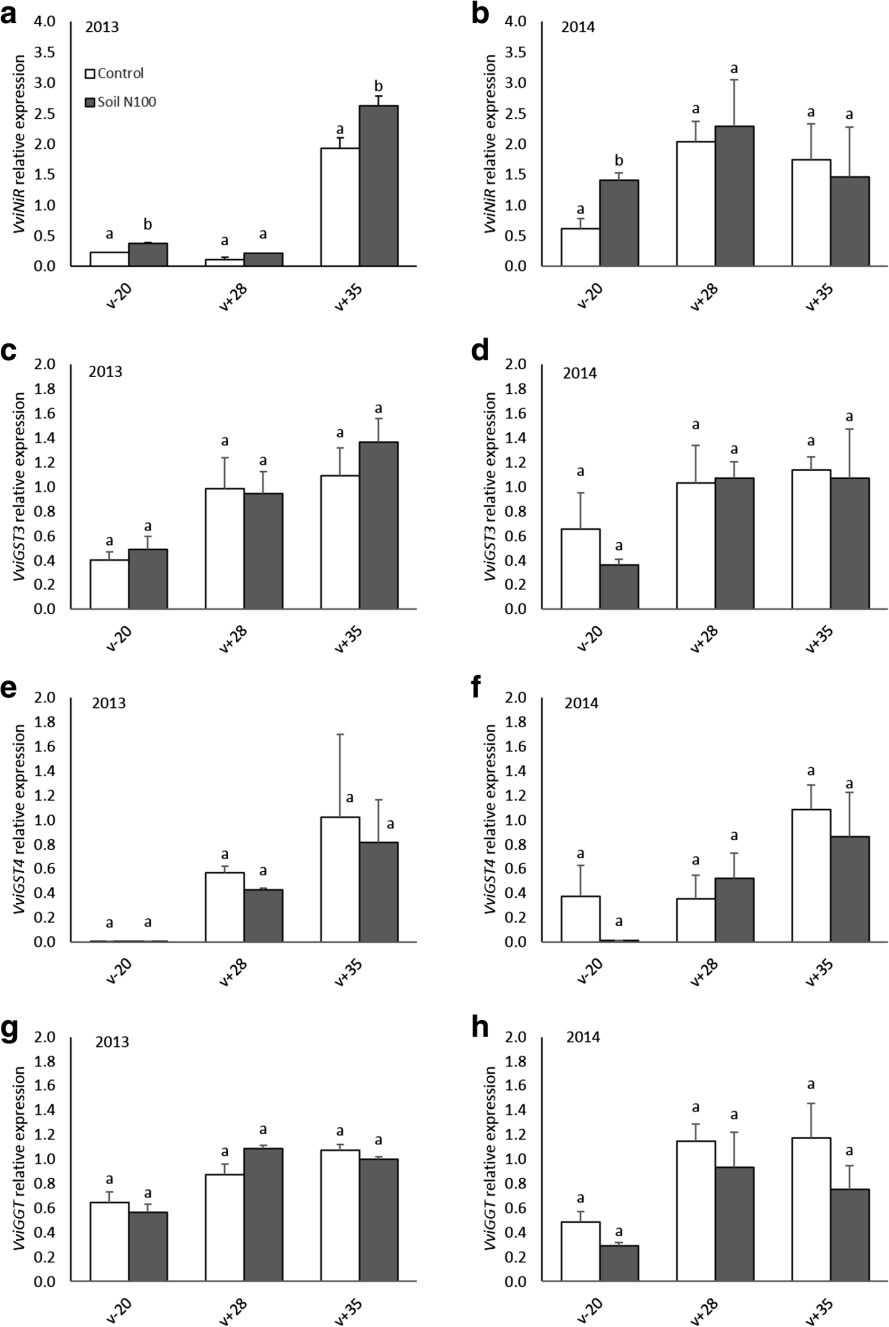
Changes in relative transcript levels of genes of *VviNiR*, *VviGST3, VviGST4* and *VviGGT* in grape berries throughout their development in the Bordeaux experimental site. Transcript levels were analyzed by real-time PCR and are shown relative to expression of *VviGAPDH* and *VviActin* in each sample. All data are presented as mean of three biological replicates and two technical replicates. Letters indicate significant differences. Error bars indicate Standard Error (SE). Statistical significance was determined by Student’s *t* test (*p* value ≤ 0.05). The white and grey bars represent the control and the soil N100 treatment respectively. (v-20), 20 days before mid-veraison; (v+28), mid-ripening; (v+35), ripeness

In a study published in 2011 [27], Kobayashi et al. described two genes, *VviGST3* and *VviGST4*, from the family of the glutathione-*S*-transferases, as essential genes in the biosynthetic pathway of 3SH. These genes are responsible for the addition of the glutathione to the *trans*-2-hexanal to produce the Glut-3SH. Another gene, *VviGGT*, responsible for the conversion of Glut-3SH to Cysgly-3SH, an intermediate precursor, was also identified [27]. The expression profile of these three genes and of *VviNiR*, a gene from the N assimilation pathway at vine roots level which serves as a positive control for vine N status [50], was determined in Sauvignon blanc berries from Bordeaux at different developmental stages (Fig. 8).

The *VviNiR* expression profile increased in 2013 during ripening and reached a maximum at (v + 35) stage. N supply resulted in an increasing of *VviNiR* transcript levels and differences were statistically significant at (v-20), an early developmental stage and at (v + 35) (Fig. 8a). In 2014, the expression of *VviNiR* increased with berry development and peaked at (v + 28). As for 2013, N increased transcript abundance of this gene mainly at (v-20) (Fig. 8b).

*VviGST3* and *VviGST4* expression profiles were also studied during grape ripening. In 2013 and 2014, for both treatments, the relative expression of *VviGST3* increased during ripening. It was 3-fold higher at (v + 35) compared to (v-20) (Fig. 8c–d). As for *VviGST3*, *VviGST4* had the same profile in 2013 and 2014 when its expression increased during the season (Fig. 8e–f). The transcript abundance of *VviGGT* was also similar in both years. It was low at (v-20) compared to the two other stages where it was to a large extent stable: the expression of *VviGGT* was 2-fold higher at (v + 35) compared to (v-20) (Fig. 8g–h). No significant differences were observed in the expression of *VviGST3, VviGST4* and *VviGGT* genes between the control and the soil N100 treatment at all stages in both years.

Furthermore, the expression profiles of these genes were also investigated in the RNA-seq data. In 2013, the expression profile of *VviNiR* (*VIT_03s0063g00370*) was modified in response to nitrogen (LFC 0.78 and 0.60 at (v + 28) and (v + 35) respectively, Additional file 1). Moreover, two other genes, a nitrate transporter-like (*VIT_01s0026g01570*) and a nitrite transporter-like (*VIT_01s0011g03400*) responded to N supply. The expression of the first one was up-regulated by this nutrient at (v + 28) and (v + 35) (LFC 0.71 and 0.92 respectively) while the expression of the second gene was down-regulated by N and it was significant only at (v + 35) (LFC − 0.63). In 2014, none of these genes were affected by N. Both in 2013 and 2014, the transcripts of *VviGST3, VviGST4* and *VviGGT* were not affected by N supply confirming results obtained by the qRT-PCR. Hence, these genes are clearly not regulated by nitrogen.

### Identification of candidate genes from the biosynthetic pathway of 3SH precursors

Only the genes differentially expressed in response to N supply were investigated, due to their potential implication in differences observed on Glut-3SH level between N treatments. Two strategies were conducted in order to identify new candidate genes from the biosynthetic pathway of 3SH precursors. The first one, a “targeted approach”, consisted in searching genes from families already described in the literature as implicated in the pathway. The second one, an “untargeted approach”, consisted in a screening of all common genes between (v + 28) and (v + 35) stages, when N effect on Glut-3SH was observed, and which are differentially expressed between the control and the soil N100 treatment.

For the “targeted approach”, we referred to the pathway already described by Kobayashi et al. [21] and Thibon et al. [22] where a glutathione-*S*-transferase (GST), a γ-glutamyltranspeptidase (GGT) and a carboxypeptidase were assumed to be essential for generating the precursors.

According to the National Center for Biotechnology Information reference sequence (NCBI RefSeq, August 03, 2015) database, 118 genes encoding GST enzymes were identified in *Vitis vinifera*. Of these genes, in 2013, 94 GST transcripts at (v + 28) and 96 at (v + 35) were detected in our RNA-seq dataset. In 2014, 97 and 102 GST transcripts were expressed at (v + 28) and (v + 35) respectively. *VviGSTs* with *p* value ≤ 0.05 and │LFC│ ≥ 0.6 were considered to respond to N supply and might be involved in differences observed on Glut-3SH level among treatments. A list of these *VviGSTs* is given in Table 4. In 2013, at (v + 28), *VIT_19s0015g02680* was 2-fold up-regulated in the soil N100 treatment compared to the control. At this same developmental stage, 2 other GSTs, *VIT_16s0039g01070* and *VIT_07s0104g01800* were 1.7-fold and 1.8-fold up-regulated in the fertilized modalities (Table 4a).

**Table 4:** Glutathione-*S*-transferase and carboxypeptidase genes differentially expressed between the control and the soil N100 treatment at mid-ripening (v + 28) or ripeness (v + 35) in (a) 2013 and (b) 2014

A			
V1_ID	Best identity description	LFC	
		(v + 28)	(v + 35)
Putative Glutathione-*S*-transferase (Bincode 26.9)			
*VIT_01s0026g02400*	GSTU10-like	ns	0.79
*VIT_06s0004g05680*	GSTU7-like	ns	0.68
*VIT_07s0104g01800*	GSTF13-like	0.87	ns
*VIT_12s0035g02100*	GSTZ2-like	ns	−0.94
*VIT_16s0039g01070*	GSTU8-like	0.79	ns
*VIT_19s0015g02680*	GSTU25-like	0.94	ns
*VIT_19s0015g02690*	GSTU25-like	ns	0.79
Putative carboxypeptidase (Bincode 29.5)			
*VIT_00s0187g00120*	Putative cysteine protease inhibitor	0.63	ns
*VIT_00s0265g00070*	Ubiquitin-protein ligase	−0.71	ns
*VIT_00s0323g00100*	Cysteine-type endopeptidase	ns	−0.61
*VIT_00s2015g00020*	F-box protein FBW2-like	1.12	ns
*VIT_00s2507g00010*	F-box protein FBW2-like	2.26	0.81
*VIT_01s0010g02860*	Mitochondrial chaperone BCS1-like	ns	−0.90
*VIT_01s0011g02000*	Serine carboxypeptidase-like 35	ns	0.71
*VIT_01s0137g00330*	Putative cysteine proteinase	ns	−0.93
*VIT_01s0150g00330*	U-box domain-containing protein 43-like	ns	0.79
*VIT_03s0088g00260*	Serine carboxypeptidase 16-like	ns	0.91
*VIT_03s0091g01290*	Serine carboxypeptidase 18-like	1.06	1.09
*VIT_04s0043g00840*	Ubiquitin-protein ligase	−0.72	ns
*VIT_05s0020g05000*	Serine-type endopeptidase inhibitor	ns	1.46
*VIT_06s0061g00710*	Zinc finger family protein	0.99	0.87
*VIT_06s0080g00150*	Serine-type endopeptidase	ns	0.73
*VIT_07s0104g00180*	Putative extracellular dermal glycoprotein	0.67	ns
*VIT_08s0007g00120*	Ubiquitin-protein ligase	0.60	ns
*VIT_08s0007g02470*	Aspartyl protease family protein	ns	0.68
*VIT_08s0040g01140*	Putative serine carboxypeptidase	1.19	ns
*VIT_08s0058g00700*	F-box family protein	ns	−0.64
*VIT_10s0116g00560*	Polyphenol oxidase	1.60	2.19
*VIT_11s0016g03010*	Peptidase family protein	ns	0.62
*VIT_13s0019g05120*	Cysteine proteinase 15A-like	ns	−0.75
*VIT_13s0047g00200*	Xylem serine proteinase 1-like	ns	0.84
*VIT_14s0006g00180*	Aspartic proteinase nepenthesin-2-like	−0.80	ns
*VIT_14s0219g00210*	Ubiquitin family protein	−0.74	ns
*VIT_15s0046g02160*	Peptidase family protein	0.64	0.66
*VIT_18s0001g00510*	Serine-type endopeptidase	ns	0.70
*VIT_18s0041g00370*	Cysteine proteinase inhibitor 1-like	0.85	ns
*VIT_19s0085g01110*	Putative aspartic proteinase	ns	1.19
B			
V1_ID	Best identity description	LFC	
		(v + 28)	(v + 35)
Putative carboxypeptidase (Bincode 29.5)			
*VIT_14s0066g01950*	Metalloendoproteinase 1-like	ns	−0.62

*p* value ≤ 0.05 and │LFC│ ≥ 0.6. *C* control. *ns* not significant

*VviGSTs* identified at (v + 35) were different from those described at (v + 28). At this stage, *VIT_01s0026g02400*, *VIT_06s0004g05680* and *VIT_19s0015g02690* were found to be up-regulated in the fertilized vines compared to the control. However, *VIT_12s0035g02100* expression profile was down-regulated by N supply (Table 4a). All identified *VviGSTs* were from the tau family (GSTU) except for *VIT_07s0104g01800* and *VIT_12s0035g02100* which are from phi (GSTF) and zeta (GSTZ) family respectively.

In 2014, no *VviGST* were differentially expressed between the control and the soil N100 treatment at both developmental stages.

The 3SH precursor pathway involves also the presence of a GGT and a carboxypeptidase. Eleven *VviGGTs* were identified in 2013 and 2014 at both stages but using the same criteria as for *VviGSTs* (*p* value ≤ 0.05 and │LFC│ ≥ 0.6), none was differentially expressed between fertilized and unfertilized vines.

Concerning the large family of carboxypeptidases, 1453 at (v + 28) and 1431 at (v + 35) were expressed in the RNA-seq data. Among these carboxypeptidases, in 2013, 15 at (v + 28) and 20 at (v + 35) had a different expression pattern among treatments. Except for 4 at (v + 28) and 5 at (v + 35), all the identified carboxypeptidases were up-regulated by N (Table 4a). In 2014, at (v + 28), none of these carboxypeptidases was regulated by vine N status. At (v + 35), only one (*VIT_14s0066g01950*) was down-regulated in response to this nutrient (Table 4b).

**Fig. 9:**
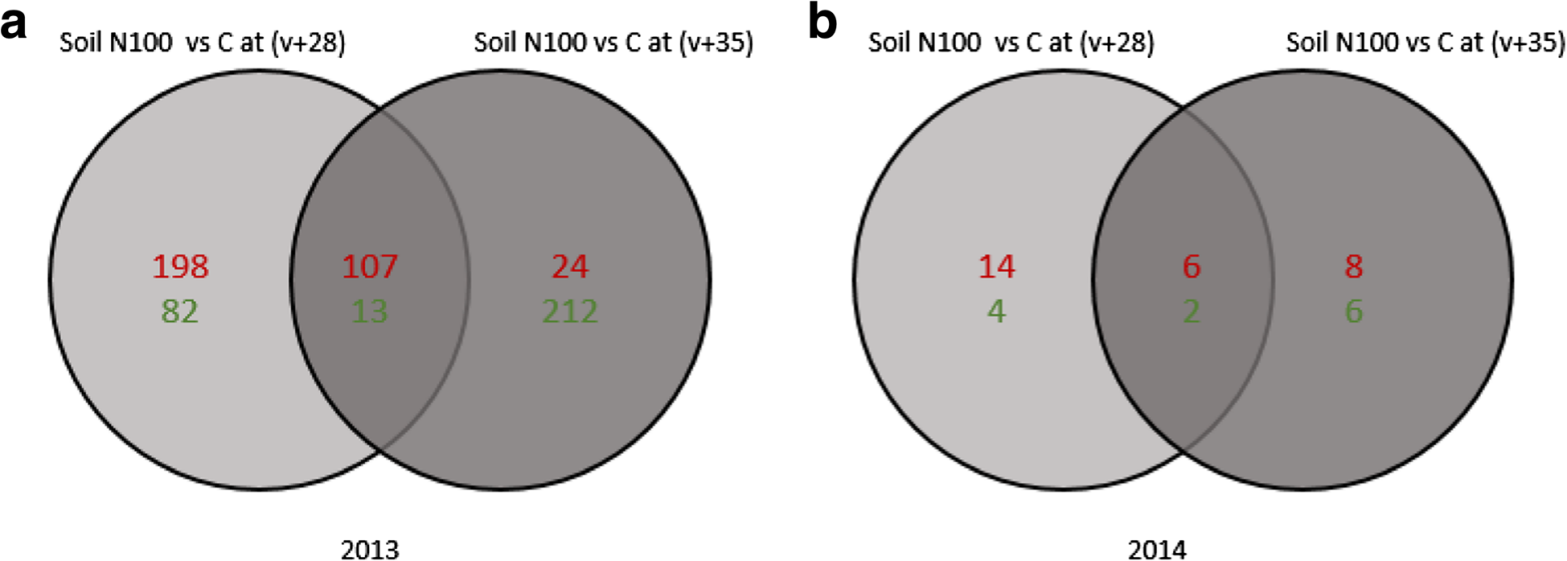
Comparison of differential gene expression between the soil N100 and the control at two developmental stages in (**a**) 2013 and (**b**) 2014. Venn diagrams indicate overlap of all differentially expressed genes obtained from each comparison between the soil N100 treatment and the control at mid-ripening (v + 28) and ripeness (v + 35). The numbers of up-regulated genes and down-regulated genes are given in *red* and in *green*, respectively. C, control

For the “untargeted method”, all genes affected by N supply at (v + 28) and (v + 35) were identified (Fig. 9). As represented in the Venn diagram, in 2013, 305 genes were up-regulated and 95 genes were down-regulated by N at (v + 28). In this category, the most represented families belong to stress, DNA, signaling, metal handling and secondary and amino acid metabolisms (Additional file 1). At (v + 35), 131 genes were stimulated by N while 225 were repressed. At this developmental stage, GO related to photosystem, secondary metabolism, cell wall, biodegradation of xenobiotics, development and DNA were the most represented (Additional file 1). Between the 2 stages, 107 common up-regulated genes and 13 down-regulated genes were identified (Fig. 9a).

In 2014, at (v + 28), 20 and six genes were up- and down-regulated by N respectively. Genes related to stress were the most represented. At (v + 35), the expression of 14 genes was stimulated by N and the expression of eight genes was repressed by this nutrient. At this stage, categories represented with the highest number of genes encompassed the stress and the secondary metabolism gene families (Additional file 2). Six up-regulated and two down-regulated genes in response to N were common between the 2 stages (Fig. 9b).

By combining the dataset from the 2 years, ten and two genes respectively were up-regulated and down-regulated by N at (v + 28). At (v + 35), five genes were up-regulated by N while one was repressed. Two up-regulated genes responding to N were common between (v + 28) and (v + 35), whereas no down-regulated genes were found in this category (data not shown). In this category, genes related to stress, hormone metabolism, RNA, protein and transport were found (Additional file 3).

We also paid special attention on genes encoding transporters given their essential role in nitrogen and secondary metabolism regulation.

In 2013, 30 and 24 genes encoding transporters responded to N supply at (v + 28) and (v + 35) respectively. They encompassed many transport process, including the transport of metals, nitrate, potassium, sulphate, calcium, sugars and amino acids. Genes involved in the transport of peptides and oligopeptides, as well as genes from the ATP binding cassette (ABC) transporters family also belonged to the transport genes differentially regulated by N. From these genes, ten were common between the two developmental stages (Table 5a). In 2014, the expression of three genes at (v + 28) and two genes at (v + 35) was modified by N fertilization. These genes are involved in metal or anions transport or belong to the ABC transporter family. Only one gene was common between the two developmental stages (Table 5b).

**Table 5:** Transporter genes differentially expressed between the control and the soil N100 treatment at mid-ripening (v + 28) or ripeness (v + 35) in (a) 2013 and (b) 2014

A			
V1_ID	Best identity description	LFC	
		(v + 28)	(v + 35)
Putative transporters (Bincode 34)			
*VIT_00s0181g00010*	Sugar transporter 1-like	ns	0.61
*VIT_00s0259g00140*	Oligopeptide transporter (AtOPT3)	ns	0.83
*VIT_01s0011g00600*	Triose-phosphate transmembrane transporter	0.73	ns
*VIT_01s0011g01280*	Cation transmembrane transporter	0.68	ns
*VIT_01s0011g03400*	Proton-dependent oligopeptide transporter (POT)	ns	−0.63
*VIT_01s0011g06000*	Vacuolar iron transporter 1	ns	−0.73
*VIT_01s0026g00270*	Potassium transmembrane transporter	0.67	ns
*VIT_01s0026g01570*	Nitrate transporter 1:2-like (AtNRT1:2)	0.71	0.92
*VIT_02s0025g00810*	Sodium hydrogen antiporter	−0.71	ns
*VIT_02s0025g00990*	ABC transporter family	0.82	1.12
*VIT_03s0017g01290*	Fatty acid transporter	−0.66	ns
*VIT_03s0017g02170*	Zinc transmembrane transporter	0.60	0.74
*VIT_03s0038g02140*	Putative amino acid permease	ns	−0.80
*VIT_03s0038g03950*	Mitochondrial substrate carrier family	−0.84	ns
*VIT_03s0063g02250*	Polyol transporter 5-like (AtPLT5)	ns	−0.83
*VIT_05s0020g03140*	Carbohydrate transmembrane transporter	0.64	ns
*VIT_05s0049g02240*	AWPM-19-like	ns	−0.70
*VIT_05s0049g02310*	Integral membrane transporter family	−0.91	ns
*VIT_06s0009g01140*	Amino acid transporter family	−0.60	ns
*VIT_06s0061g00440*	Heavy-metal-associated domain-containing protein	0.68	ns
*VIT_06s0061g00730*	Gamma tonoplast intrinsic protein (Gamma-TIP)	1.32	1.01
*VIT_07s0151g00340*	Sulfate transporter 3;1	ns	1.07
*VIT_08s0007g02240*	Cation exchanger 3	0.73	ns
*VIT_08s0007g04780*	Gamma tonoplast intrinsic protein (Gamma-TIP)	0.77	0.71
*VIT_08s0007g06760*	Putative metal tolerance protein (MTPc3)	0.66	ns
*VIT_10s0003g02540*	P-Glycoprotein 9	ns	−0.77
*VIT_10s0003g02620*	AWPM-19-like membrane family	−0.66	ns
*VIT_10s0003g05480*	Phosphoglyceride transfer family protein	1.00	1.49
*VIT_11s0016g04160*	Sulfate transporter 3;5 (SULTR3;5)	0.71	0.70
*VIT_13s0019g04220*	Tryptophan/tyrosine permease family	−0.63	ns
*VIT_13s0019g04660*	Amino acid transporter family	1.22	1.31
*VIT_13s0067g02220*	Amino acid transporter family	0.67	ns
*VIT_14s0030g00340*	Sugar transporter family	ns	0.60
*VIT_14s0068g02190*	Chloride channel protein	1.23	0.89
*VIT_14s0108g00430*	Auxin efflux transporter	ns	0.65
*VIT_14s0108g00630*	Amino acid transporter family	1.29	ns
*VIT_15s0046g02420*	Plasma membrane intrinsic protein (PIP1A)	0.68	ns
*VIT_16s0050g01620*	ABC transporter family protein	0.73	ns
*VIT_18s0001g02120*	Inorganic anion transmembrane transporter	ns	−0.73
*VIT_18s0001g04910*	Sulfate transmembrane transporter (SULTR1;3)	0.64	ns
*VIT_18s0041g00590*	Proton-dependent oligopeptide transporter (POT)	ns	0.87
*VIT_19s0015g00860*	Oligopeptide transporter-like (AtOPT1)	−1.12	−0.96
*VIT_19s0090g01480*	Sodium transporter	ns	−0.62
B			
V1_ID	Best identity description	LFC	
		(v + 28)	(v + 35)
Putative transporters (Bincode 34)			
*VIT_01s0010g02440*	Chlorophyll/glutathione-S-conjugate transporter ATPase (AtMRP3-like)	1.08	ns
*VIT_14s0068g02190*	Chloride channel protein	0.90	ns
*VIT_18s0001g02140*	Manganese ion transmembrane transporter	−0.94	ns
*VIT_19s0085g00740*	Zinc transmembrane transporter	0.91	1.24

*p* value ≤ 0.05 and │LFC│ ≥ 0.6. *C* control, *ns* not significant

Only genes common to both stages that were differentially expressed between the control and the N100 treatment were examined due to the fact that Glut-3SH level was modified at both stages. Within this category, in 2013, genes encoding a putative nitrate transporter (*VIT_01s0026g01570*), a putative zinc transporter (*VIT_03s0017g02170*), a putative sulfate transporter (*VIT_11s0016g04160*) and a putative chloride transporter (*VIT_14s0068g02190*) were found. Two gamma tonoplast intrinsic protein (*VIT_06s0061g00730* and *VIT_08s0007g04780*), a putative phosphoglyceride transporter (*VIT_10s0003g05480*) and a putative amino acid transporter (*VIT_13s0019g04660*) were also identified. Interestingly the expression of genes encoding an ABC transporter (*VIT_02s0025g00990*) and an oligopeptide transporter (OPT, *VIT_19s0015g00860*) was also differentially expressed between treatments. The expressing of all these genes was up-regulated by N except for the OPT gene which was down-regulated. It was 2-fold lower in the soil N100 treatment compared to the control. In addition to *VviNiR*, *VviGST3*, *VviGST4* and *VviGGT* which the expression validate the RNA-seq results, the expression of *VIT_19s0015g00860* (*VviOPT*) was confirmed by real-time PCR. It was significantly down regulated by N at (v + 35) in 2013 and at (v + 28) in 2014 (Additional file 4).

In 2014, only a putative zinc transporter (*VIT_19s0085g00740*) was common between the two developmental stages.

## Discussion

The aim of this study was to investigate the effect of vine N status on the content of two major volatile thiols, 4MSP and 3SH, and the accumulation of the precursors of 3SH in grape berries and must. The implication of *VviGST3*, *VviGST4* and *VviGGT* genes in the biosynthetic pathway of 3SH precursors was also assessed and new candidate genes which might be involved in its genesis were identified.

A complete agronomic approach was set up. Vines were the subject to various levels of N status, while vine water status and vigor were controlled. This experimental set-up allowed us to study the direct effect of N nutrition on the targeted metabolites, without an interference with other parameters. The agronomic approach was complemented with an analytical approach where 3SH and its precursors were quantified and with a global transcriptomic approach. This multi-approach study allowed a better understanding of the phenomena and mechanisms underlying the regulation of volatile thiol and volatile thiol production by vine N status.

In the wine, 3SH increased with vine N status. Differences were statistically significant in the majority of combinations. In berries, Glut-3SH content was very low in early stages of development and increased during ripening regardless of vine N status, in accordance with Roland et al. and Cerreti et al. [51, 52]. The increase was in general greater in N+ conditions compared to the control particularly at mid-ripening and ripeness stages. Cys-3SH was detectable only in Bordeaux and Sancerre berries in 2013. Its concentration was stable during berry ripening and its synthesis was not impacted by vine N status. Like in the berry, Glut-3SH content in must was affected by vine N status while Cys-3SH did not reveal any response to N supply. Both precursors are supposed to be implicated in the genesis of 3SH and until recently, the Cys-3SH was considered as the major precursor used by the yeast to release the 3SH. In the present study, N addition did not affect Cys-3SH level while it impacted the content of Glut-3SH and consequently the content of 3SH. This observation seems to indicate that 3SH could be synthetized from Glut-3SH, independently from Cys-3SH. This result is consistent with Cordente et al. [53], who showed that Glut-3SH can be a direct precursor of 3SH. Still, further studies are required to elucidate the exact pathways for the release of this thiol (i.e. from C6 compounds…) [54–56].

Precursor synthesis was higher in the must compared to berries. As berries were immediately frozen at sampling, precursors synthesis and enzyme activity in the berry were blocked. In must, enzyme activity was maintained, and they were in direct contact with the substrate. This could explain the higher concentrations of precursors in the must, in addition to a putative effect of other pre-fermentation operations. Moreover gene expressions were stimulated by high vine N-status, enhancing precursors synthesis. More investigations are required to obtain a precise explanation of the striking differences in concentration of precursors between berries and must.

The response of vine to N status in terms of metabolite synthesis or accumulation in the berries and genes profiling was relatively different between 2013 and 2014. This maybe due to the different plant material used in both years (clone 316 grafted on Fercal in 2013 and clone 108 grafted on 161-49 C in 2014) or to differences in climatic conditions, 2014 being warmer year and 2013 a year with more sunshine hours during July and August (data not shown). N absorption and/or assimilation can be influenced by the clone [57] or by the rootstock [38]. In Bordeaux in 2014, the maximal accumulation of Glut-3SH in the berries was reached at (v + 28) while in 2013, it was attained at (v + 35) with concentrations 2-fold higher for the soil N100 treatment. Moreover, in 2013, more genes were differentially expressed between the two treatments compared to 2014 at both developmental stages indicating that the response of grapevine to environnemental conditions also depend on the genotype of the plant.

In 2011, Kobayashi et al. [27] demonstrated that *VviGST3*, *VviGST4* and *VviGGT* are key genes in the biosynthetic pathway of 3SH precursors and that their expression is correlated to Glut-3SH and Cys-3SH levels in grape berries. Higher transcript abundance of *VviNiR* in the berries were observed in soil N100 treatment, confirming that the vines responded to N supply at a transcriptomic level in our study. Unlike *VviGST3*, *VviGST4* and *VviGGT* whose transcript abundances were similar in vines with highly different N status, Glut-3SH content was influenced by N supply. Thus, in the present study, *VviGST3* or *VviGST4* transcript abundance do not relate to Glut-3SH contents. Hence, it can be assumed that these two genes are not responsible of the variation observed in Glut-3SH levels in berries. The mode of genesis of the different precursors and the potential implication of a *VviGST* or a *VviGGT* needs further investigation.

In plants, the glutathione-*S*-transferases are a superfamily of proteins involved in enzymatic detoxification of endo- and xenobiotics. Plant GSTs are divided into six sub-classes namely tau (U), phi (F), zeta (Z), theta (T), lambda (L) and dehydroascorbate reductases [58]. In *A. thaliana*, AtGSTU25 (GenBank: 838289) was demonstrated to bind to *S*-hexanol-glutathione which can be the product of the addition of glutathione to *trans*-2-hexenal [59–61]. These compounds serve as precursors of the 3SH in the grape berries. Among the identified GSTs in 2013, *VIT_19s0015g02680* possesses a predicted protein sequence which presents 69 % of similarity with that of *AtGSTU25*. Taken all together, *VIT_19s0015g02680* could be a potential candidate gene encoding an enzyme implicated in the biosynthetic pathway of 3SH precursors.

In the present study, *VviGGT* transcript abundance was not affected by vine N status, and likewise, the level of Cys-3SH, the metabolite supposed to be formed in response to its enzymatic activity. The expression of *VviGGT* gene was not regulated by N, which may explain the absence of an effect of N on the cysteinylated precursor.

The global transcriptomic approach allowed the identification of genes encoding transporters involved in peptides and oligopeptides transport. In *A. thaliana*, based on sequence similarity and mechanism, three gene families have been shown to transport peptides: (i) the ATP-binding cassette (ABC) superfamily, (ii) the peptide transporter (PTR) or the proton–dependent oligopeptide transporter (POT) family and (iii) the oligopeptide transporter (OPT) family [62].

In this work, an OPT gene (*VIT_19s0015g00860, VviOPT1*) was identified. In *Vitis vinifera*, 18 OPT genes were identified that possess a conserved OPT domain essential for their transporter activity [63]. The OPT proteins may be involved in 4 different processes: long-distance metal distribution [64], heavy metal sequestration [65, 66], nitrogen mobilization [62, 66] and glutathione transport [65–68]. The protein sequence of *VviOPT1* possesses 47.9 % of similarity with that of *ScOPT1* in *S. cerevisiae* (GenBank: 853218). Subileau et al. (2009) showed that the knockout mutant of the *OPT1* gene in *S. cerevisiae* reduced the accumulation of 3SH precursors in grape must. They deduced that the majority of Glut-3SH is transported into yeast via OPT1. In the absence of this transporter, the precursor uptake limited significantly the production of 3SH [55]. Recently, Santiago and Gardner [69] demonstrated that ScOPT1 is required to the conversion of Glut-3SH to 3SH. Noteworthy is the fact that *VviOPT1* expression profile was also affected by N at (v + 35) stage in 2014 (LFC − 0.71 and *p* value 0.005), however the adjusted *p* value was above threshold.

Concerning the ABC transporter genes, *VIT_02s0025g00990* and *VIT_16s0050g01620* responded to N supply at both stages in 2013. In 2014, another ABC transporter (*VIT_01s0010g02440*) responded to this nutrient but its expression was only significantly modified at (v + 28). In *A. thaliana,* ABC transporters could be involved in glutathione-*S*-conjugates and chlorophyll catabolism [70].

Among the POT family, *VIT_01s0011g03400* which is responsive to N status, encodes a nitrite transporter similar to a putative transporter involved in nitrite metabolism (73 % of similarity) described in *A. thaliana* (AT1G68570; GenBank: 843186).

Based on these evidences, the *VviOPT* genes and the *ABC transporter* genes identified in the present study may be potential candidate genes implicated in the transport of GSH or the GSH-*S*-conjugates that are necessary for the genesis of 3SH precursors.

In Bordeaux, a significant positive effect of N supply on Glut-3SH level was observed in 2013 and 2014 while in Sancerre the impact was non-significant. In the Sancerre vineyard in both vintages, vine vigor was increased in soil N100 treatment. The non-significant response of Glut-3SH level to N nutrition in Sancerre may be due to a decrease in 3SH precursors synthesis in soil N100 treatment, related to increased vine vigor. Greater leaf areas might have modified the microclimatic conditions in the bunch zone, decreasing the temperature and radiation at the grape level, although no precise micro climatic measurements were carried out. The observed effect could be due either to indirect UV–B or temperature differences. Indeed, an effect of harvest temperature on the concentration of 3SH precursors was described by Kobayashi et al. [27], suggesting that the accumulation of these molecules depend on the climatic conditions. Light or radiation may stimulate the synthesis of thiol precursor or enhance their degradation. No studies have been published on the direct effect of temperature and radiation on 3SH precursors, and more research is required on this highly relevant subject. So the lack of impact of N on Glut-3SH in the Sancerre experiment might be the result of a modification of the microclimate at the level of bunch zone.

The positive effect of nitrogen on 3SH content in wine could also result from a stabilisation of this compound in berries from high N vines. Thiols are very sensitive molecules and can be lost due to oxidation reactions in the grape must [71–73]. The presence of antioxidant molecules in wines from soil N100 modality might explain their stabilization. Among these antioxidants, glutathione (GSH) plays an important role in the stability of volatile thiols in wine. This molecule is very important in oenology, especially for the production of white wine. GSH content responds positively to N status [28, 29] and its implication in the biosynthetic pathway of 3SH was recently demonstrated [22]. The role of glutathione on the synthesis or stability of volatile thiol, in particular with regard to 3SH, requires further research. It needs to be clarified if higher 3SH levels in wine in the presence of high GSH levels results from increased production or greater stability of 3SH.

## Conclusion

The effect of vine N status on volatile thiols in wines from *Vitis vinifera* cv Sauvignon blanc wines and their precursors in grapes and must was investigated combining agronomic, analytic and transcriptomic approaches. An effect of vine N status on 3SH was found, while N did not have an effect on 4MSP. High vine N status increased 3SH level in wine through an increase of Glut-3SH content in grape berries and must, but did not impact Cys-3SH level. This observation seems to indicate that 3SH could be synthetized from Glut-3SH, independently from Cys-3SH and from the activity of VviGST3, VviGST4 and VviGGT. In the berry, the impact of N on Glut-3SH involves putatively another type of GSTs, as well as oligopeptide transporters.

### Sample collection in a commercial vineyard

Samples were collected for this study at the commercial vineyard of ChâteauCouhins (Pessac-Léognan, France) and Domaine Fontaine-Audon (18240 Sainte-Gemme en Sancerrois, France) with the authorization of the owners and following the INRA’s ethics charter (http://institut.inra.fr/en/Objectives/Promoting-ethics-and-a-code-of-conduct/All-reports/Ethics-Charter/Professional-Ethics-Charter).

## Abbreviations

3SH, 3-sulfanylhexan-1-ol; 4MSP, 4-methyl-4-sulfanylpentan-2-one; 6SH, 6-sulfanylhexanol; ABC, ATP-binding cassette; Cys-3SH, *S*-3-(hexan-1-ol)-cysteine; Cys-4MSP, *S*-4-(4-methylpentan-2-one)-L-cysteine; Cysgly-3SH, *S*-3-(hexan-1-ol)-cysteinylglycine; GGT, γ-glutamyltranspeptidase; Glut-3SH, *S*-3-(hexan-1-ol)-glutathione; Glut-4MSP, *S*-4-(4-methylpentan-2-one)-L-glutathione; GO, gene ontology; GSH, glutathione; GST, glutathione-*S*-transferase; LFC, log_2_(Fold-Change); LOQ, limit of quantification; MMSB, 4-methoxy-2-methyl-2-sulfanylbutan; N, nitrogen; NiR, nitrite reductase; OPT, oligopeptide transporter; POT, proton-dependent oligopeptide transporter; PTR, peptide transporter; RPKM, reads per exon kilo base per million mapped sequence reads; Soil N100, 100 kg per hectare of nitrogen applied to the soil; v, mid-veraison; v + 28, mid-ripening; v + 35, ripeness; v-20, bunch closure; YAN, yeast available nitrogen

## Additional files

Additional file 1: Genes differentially expressed in response to N at (v + 28) and (v + 35) in 2013. *p* value ≤ 0.05 and │LFC│ ≥ 0.6. C, control; ns, not significant. (XLSX 49 kb) Additional file 2: Genes differentially expressed in response to N at (v + 28) and (v + 35) in 2014. *p* value ≤ 0.05 and │LFC│ ≥ 0.6. C, control; ns, not significant. (XLSX 12 kb) Additional file 3: Genes differentially expressed in response to N at (v + 28) and (v + 35) in 2013 and 2014. *p* value ≤ 0.05 and │LFC│ ≥ 0.6. C, control; ns, not significant. (XLSX 10 kb) Additional file 4: Changes in relative transcript levels of *VIT_19s0015g00860* (*VviOPT*) in grape berries at mid-ripeness (v + 28) and ripeness (v + 35) in the Bordeaux experimental site. Transcript levels were analyzed by real-time PCR and are shown relative to expression of *VviGAPDH* and *VviActin* in each sample. All data are presented as mean of three biological replicates and two technical replicates. Letters indicate significant differences. Error bars indicate Standard Error (SE). Statistical significance was determined by Student’s *t* test (*p* value ≤ 0.05). (DOCX 30 kb)
