# Supplementary figures and images for: Vine nitrogen status and volatile thiols and their precursors from plot to transcriptome level
Source: BMC Plant Biol. 2016 Aug 8;16:173. doi: 10.1186/s12870-016-0836-y (PMC4976470; doi:10.1186/s12870-016-0836-y)

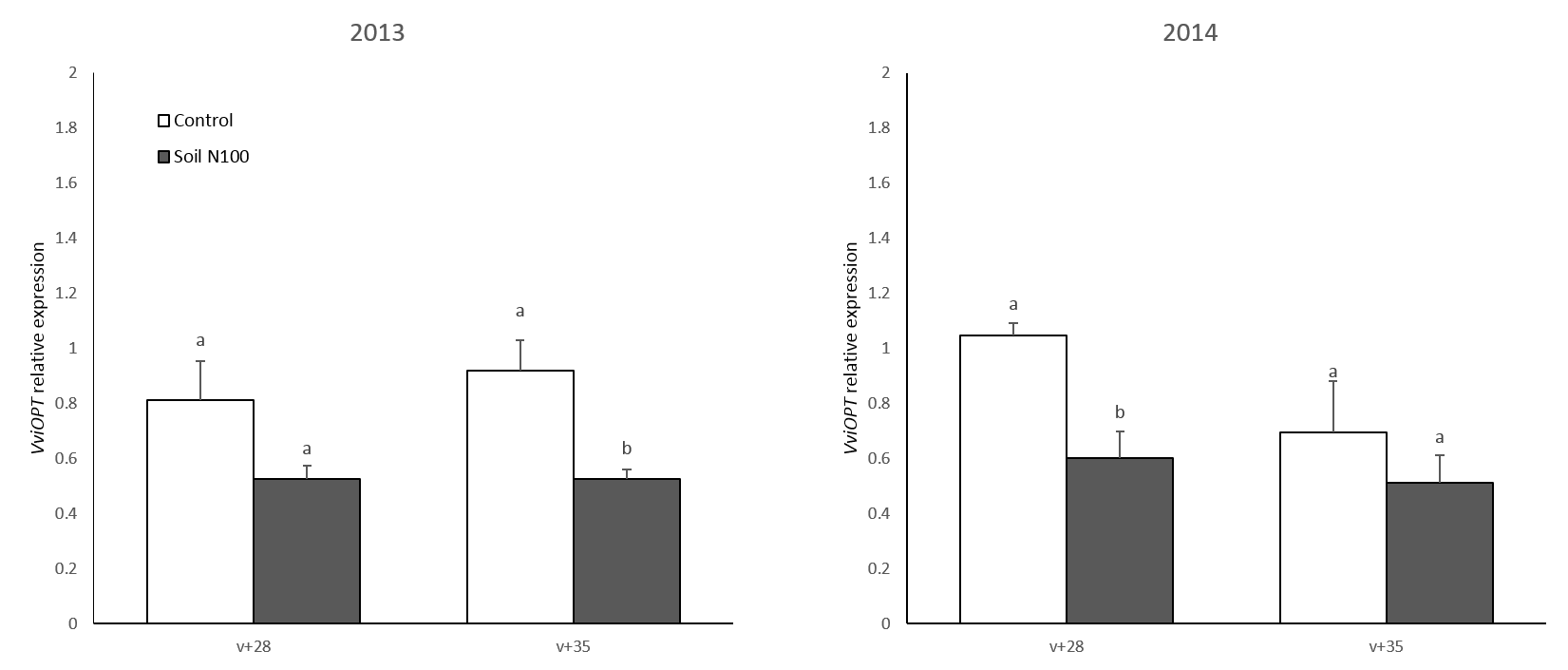


**Additional file 4.**

Supplement: Additional file 4: — Changes in relative transcript levels of VIT_19s0015g00860 (VviOPT) in grape berries at mid-ripeness (v + 28) and ripeness (v + 35) in the Bordeaux experimental site. Transcript levels were analyzed by real-time PCR and are shown relative to expression of VviGAPDH and VviActin in each sample. All data are presented as mean of three biological replicates and two technical replicates. Letters indicate significant differences. Error bars indicate Standard Error (SE). Statistical significance was determined by Student’s t test (p value ≤ 0.05). (DOCX 30 kb) [file 12870_2016_836_MOESM4_ESM.docx]
